# Mapping RNA–capsid interactions and RNA secondary structure within virus particles using next-generation sequencing

**DOI:** 10.1093/nar/gkz1124

**Published:** 2019-12-04

**Authors:** Yiyang Zhou, Andrew Routh

**Affiliations:** 1 Department of Biochemistry and Molecular Biology, The University of Texas Medical Branch, Galveston, TX, USA; 2 Sealy Center for Structural Biology and Molecular Biophysics, University of Texas Medical Branch, Galveston, TX, USA

## Abstract

To characterize RNA–capsid binding sites genome-wide within mature RNA virus particles, we have developed a Next-Generation Sequencing (NGS) platform: viral Photo-Activatable Ribonucleoside CrossLinking (vPAR-CL). In vPAR-CL, 4-thiouridine is incorporated into the encapsidated genomes of virus particles and subsequently UV-crosslinked to adjacent capsid proteins. We demonstrate that vPAR-CL can readily and reliably identify capsid binding sites in genomic viral RNA by detecting crosslink-specific uridine to cytidine transitions in NGS data. Using Flock House virus (FHV) as a model system, we identified highly consistent and significant vPAR-CL signals across virus RNA genome, indicating a clear tropism of the encapsidated RNA genome. Certain interaction sites coincide with previously identified functional RNA motifs. We additionally performed dimethyl sulfate mutational profiling with sequencing (DMS-MaPseq) to generate a high-resolution profile of single-stranded genomic RNA inside viral particles. Combining vPAR-CL and DMS-MaPseq reveals that the predominant RNA–capsid interaction sites favored double-stranded RNA regions. We disrupted secondary structures associated with vPAR-CL sites using synonymous mutations, resulting in varied effects to virus replication, propagation and packaging. Certain mutations showed substantial deficiency in virus replication, suggesting these RNA–capsid sites are multifunctional. These provide further evidence to support that FHV packaging and replication are highly coordinated and inter-dependent events.

## INTRODUCTION

Icosahedral RNA viruses must package their genetic cargo into the restrictive and tight confines of the protected virions. High resolution structures of RNA viruses have been solved by Cryo-EM and crystallography, but the encapsidated RNA often eluded visualization due to the icosahedral averaging imposed during image reconstruction. Asymmetrical reconstructions of some icosahedral RNA virus particles have revealed that the encapsidated RNAs conform to specific structures (1–3), which may be related to programmed assembly pathway or an energy-minima for RNA folding during or after encapsidation (4). Despite these advances, determining whether encapsidated RNA genomes conform to a single structure and what regions of the viral RNA genome interact with the inner surface of the capsid shell remain challenging. Furthermore, for many viral systems it remains to be determined whether there exists a single RNA structure with conserved topology in RNA virus particles or an ensemble of genomic RNA structures. This is important as resolving these features will inform on the elusive structures of the asymmetrically encapsidated genomic material and how virus particles are assembled.

Flock House virus (FHV) is a non-enveloped, single-stranded positive-sense RNA (+ssRNA) virus from the family *Nodaviridae*. The small bipartite genome comprising RNA 1 (3.1 kb) and RNA 2 (1.4 kb) is packaged into a 34 nm non-enveloped T = 3 icosahedral virion. Only two non-structural proteins are produced by FHV RNA 1: the RNA-dependent RNA polymerase (RdRp) and sub-genomic RNA encoded protein, B2. The B2 protein was discovered as the virus's approach to evade the invertebrate anti-viral RNA silencing machinery (5,6), which thereafter led to the discovery of similar mechanism in plant cells (7). FHV RNA 2 encodes the structural protein for virus assembly. A precursor protein α (43 kDa) assembles into a non-infectious provirion (8,9). The mature, infectious virions are derived from an autocatalytic process of provisions, where protein α cleaves into proteins β (38 kDa) and γ (5 kDa) (8,10). FHV is perhaps the best studied *alphanodavirus* and provides a powerful model system by virtue of its small genome size (4.5 kb), genetic tractability and ability to infect Drosophila and mosquito cells in culture and insects (reviewed in (11,12)). More recently, FHV has been adapted into the medical field. FHV-related vaccine developments utilized either the viral particle as antibody-display system (13) or the viral RNA as trans-encapsidated chimeric viral vaccine platform (14–16).

Both authentic virions of FHV and the related Pariacoto virus have been reconstructed by cryo-EM and X-ray crystallography to reveal highly ordered dodecahedral cages of RNAs (17,18). The X-ray structure of FHV virion showed electron density at the icosahedral 2-fold axis, which was modelled as an ordered RNA duplex of ∼20 nucleotides (19). This would account for 1800nts (more than one third) of the viral genome, implicating a highly-ordered and specific set of interactions between the viral protein capsid and the encapsidated genome. Interestingly, recombinantly expressed virus-like particles (VLPs) of FHV also exhibit a similar dodecahedral RNA cage despite packaging predominantly cellular RNAs. This indicates that viral capsid may either impose structure upon the encapsidated RNA or select for natively structured host RNAs such as ribosomal RNAs (20,21). However, as these structures are obtained with icosahedral averaging, we still do not know what regions or sequences of viral genomic RNA comprise the RNA cage.

The FHV encapsidation process remains largely unknown. One molecule of each RNA 1 and 2 is specifically encapsidated into virus particles (22), while subgenomic RNA 3 is excluded (23). Several components of the capsid protein such as the arginine–rich motif and the C-terminal FEGFGF motif have been demonstrated to be essential determinants of packaging specificity of RNA 1, RNA 2 or both (24–26). It was also speculated that FHV packaging process may be in close association with viral replication and/or translational events (27–30). In the virus genome, one stem-loop structure in RNA 2 proximal to 5′ end was demonstrated to be required for RNA 2 packaging (31). However, it remains unclear whether there are similar packaging sites on RNA 1 or 2, and how these sites interact and thus recruit capsid protein to fulfill virus encapsidation.

Next-generation sequencing (NGS) in combination with crosslinking techniques provides a high-throughput approach to study transcriptome-wide RNA-protein interactions (reviewed in (32)). A number of new technologies have successfully described interactions between RNA-binding proteins (RBPs) and different types of RNAs, including nascent transcripts, mRNAs, microRNAs and ribosomal RNAs. Among these, PAR-CLIP (Photoactivatable Ribonucleoside-Enhanced Crosslinking and Immunoprecipitation) (33) utilizes a 365 nm UVA-activatable ribonucleoside analog 4-thiouridine (4SU) to effectively crosslink RNA to bound proteins. The enriched crosslinked RNAs result in a highly specific U to C transition during NGS library preparation (34–36), granting the ability to rapidly identify RBP and microRNA target sites on a transcriptomic scale (33).

In an analogous fashion to PAR-CLIP, here we applied the same principle to study the interaction of FHV genomic viral RNA in the context of assembled virions. Unlike the complex cellular micro-environment, virions represent a highly simplified enclosure with few well-defined components (viral RNA and capsid). Therefore, we were able to identify for specific RNA–capsid interaction events in virions without interference from other cellular components. Furthermore, since viruses can be readily separated from other cellular components, we avoided the need of immunoprecipitation for RNA recovery, and thus largely simplifying the PAR-CLIP methodology. This method is hence named *‘vPAR-CL’ (viral PhotoActivatable-Ribonucleoside-enhanced CrossLinking)*.

Here, using FHV as a model system, vPAR-CL methodology was validated by determining that the increased U to C (U–C) transition rate was highly specific to crosslink between viral RNA and capsid. We noticed that the intensity of vPAR-CL signals was subjected to the dose of 4SU and time of incubation. Triplicate FHV vPAR-CL experiments revealed significant and highly consistent vPAR-CL signals across the encapsidated genome, which implicates a clear tropism of RNA cage inside capsid shell. The multiple clusters of vPAR-CL sites suggest that FHV encapsidation may require multiple synergetic packaging sites. DMS-MaPseq (dimethyl sulfate mutational profiling with sequencing) was used to chemically probe single-stranded FHV genomic RNA in virions. We thus constructed a whole genome DMS-MaPseq-imposed RNA secondary structure map for FHV. We noticed RNA–capsid interaction sites favored double stranded RNA regions. Synonymous mutations were designed to disrupt predicted vPAR-CL sties in dsRNA regions, which resulted in varied effects to virus RNA replication, propagation, and virulence. Mutations over certain vPAR-CL sites showed evidential deficiency in RNA replication, suggesting these sites serve a multifunctional role in both virus packaging and replication. This provides further evidence to support that FHV packaging and replication are highly synchronized and inter-dependent events.

## MATERIALS AND METHODS

### Cell culture and virus

*Drosophila melanogaster* (S2) cells were regularly maintained and passaged with Schneider's *Drosophila* Media (Gibco) containing 10% fetal bovine serum, 1 × Antibiotic-Antimycotic (Gibco), 1 × MEM non-essential amino acids solution, and 1 mM sodium pyruvate.

As described previously (37), wild-type (wt) Flock House virus (FHV) was generated by transfecting S2 cells with pMT plasmid vectors (Invitrogen) containing respective genomes (NC_004146 for RNA 1, and NC_004144 for RNA 2). Copper sulfate was used to induce the promoter 24 h post transfection, while viruses were allowed to accumulate until 3 days post induction to yield passage 0 (P0) virus/cell mixture. The P0 transfected cells and viruses were then used to inoculate naïve S2 cells in a T75 flask for 3 days to yield passage 1 viruses, which were purified and used as FHV inoculum in this study, unless otherwise mentioned. All virus transfections, infections, and passages with S2 cell culture were maintained in a 27°C incubator.

To purify FHV, 1% Triton X-100 was added to the cell culture containing P1 viruses. Cell culture underwent one freeze-thaw cycle, and cell debris was removed with 3000 × g centrifugation. FHV in the supernatant was crudely purified with 4% polyethylene glycol (PEG) 8000 and centrifuged (6000 × g) to remove debris (15). This was followed by DNase I and RNase A overnight digestion, to remove any co-precipitated cellular DNA or RNA. Unless otherwise mentioned, viruses were further purified with a 10–40% sucrose gradient, and ultracentrifuge at 40 000 RPM for 1.5 h. Viruses were then concentrated with 100K MWCO polyethersulfone (PES) membrane protein concentrator (Pierce) and washed three times with 10 mM Tris pH 7.4.

### vPAR-CL and ClickSeq

S2 cells were maintained in T75 flask until 70–90% confluency. Cells were infected with purified Flock House virus (P1) at MOI = 1 (37–39). As an initial dose, 4-thiouridine (Sigma-Aldrich) was supplemented to the cell culture to 100 μM as 1× concentration with virus. An optional ‘boost’ dose of 4-thiouridine can also be supplied 16 h post infection (Figure 3A). Cells and viruses were harvested at 16 or 40 h post infection (Figure 3A). Viruses were purified with methods described above.

UV crosslinking was conducted at 4°C in dark room. The nuclease-treated and purified 4SU-containing viruses were placed uncovered over ice and irradiated with 0.15 J/cm^2^ (33,40) of 365 nm UV light (3UV-38, UVP). After crosslinking, viruses were digested with 8U of proteinase K (NEB) at 37°C for 30 min. Crosslinked RNAs were extracted and purified with RNA Clean & Concentrator (Zymo Research) to yield RNA template for 4SU+/UV+ sequencing library sample. Unless otherwise mentioned, the same 4SU-containing virus without any UV irradiation was prepared in the same way to give RNA template for 4SU+/UV- control library.

Both the crosslinked and uncrosslinked viral RNA were used to construct the ClickSeq libraries per standard ClickSeq method, which is detailed previously (37,41,42). 250ng of RNA template was used in reverse transcription reaction with 1:35 Azido-NTPs:dNTPs ratio and SuperScript III reverse transcriptase (Invitrogen). Equal molar of each indexed library was pooled and run on a HiSeq 1500 platform (Illumina), with single read rapid run flowcell for 1 × 150 reads and seven nucleotides for the index.

### DMS-MaPseq and ClickSeq

Dimethyl sulfate (DMS) RNA methylation method was described previously (43,44). In this study, nuclease-treated and purified FHV virions were supplemented with DMS to 5% final concentration. After 5 min incubation at 30°C, reaction was quenched on ice for 5 min with 2 volumes of 10 mM Tris pH 7.4 and 30% 2-mercaptoethanol (BME). RNA extraction was conducted with Quick-RNA Viral Kits (Zymo Research) with additional BME in the extraction buffer. The untreated (DMS-) control sample comprises the same virus stock with identical treatments as above, but without DMS supplementation.

Methylated FHV RNAs and respective controls were processed with ClickSeq library construction method as describe above with the exception of the use of the high-fidelity and processive thermostable group II reverse transcriptase enzyme (TGIRT-III, InGex) during reverse transcription. 100 U of TGIRT-III was mixed with 250 ng of RNA template, 0.5 mM of AzNTPs/dNTPs mixture (AzNTPs:dNTPs = 1:35), and the following reaction conditions: 5 mM dithiothreitol (Invitrogen), 10 U RNaseOUT (Invitrogen), 50 mM Tri–HCl pH 8.0, 75 mM KCl and 3 mM MgCl_2_. The reaction mix was incubated at room temperature for 10 min, followed by 57°C incubation for 1.5 h, and 75°C termination for 15 min. The terminated reaction was then digested with RNase H to remove RNA template. The purified cDNA was click-ligated with Illumina adapters and final PCR amplification with indexes. Library pooling and Illumina sequencing platform are the same as above.

### Bioinformatics and data analysis

The Illumina sequencing data of both vPAR-CL and DMS-MaPseq were subjected to the following bioinformatic pipelines. First, the Illumina sequencing adapter sequence ‘AGATCGGAAGAGC’ was trimmed with *cutadapt* (45) (command line parameters: -b AGATCGGAAGAGC -m 40); then, we used *FASTX toolkit* (http://hannonlab.cshl.edu/fastx_toolkit/index.html) to remove the remaining random nucleotides from the Illumina adapter sequence and random base-pairing as a result of azide-alkyne cycloaddition from cDNA fragments (command line parameters: fastx_trimmer -Q33 -f 7). A further quality filter was applied to remove any reads that contained more than 4% nucleotides with a PHRED score <20 (command line parameters: fastq_quality_filter -Q33 -q 20 -p 96). The remaining reads were aligned to FHV genome (NC_004146, and NC_004144). Data generated from vPAR-CL experiments were aligned for end-to-end matches with *Bowtie (v1.0.1)* (46) (command line parameters: -v 2 –best). Data generated from DMS-MaPseq experiment were aligned with *Bowtie2* (47) to allow gapped alignments (command line parameters: –local). The aligned reads were binarily converted, merged, indexed, and sorted using *SAMtools* (48).

For both vPAR-CL and DMS-MaPseq, we excluded any nucleotide location with <10k coverage to ensure reliable transition rate calculation (an example of coverage map can be found in Supplemental Figure S4). For vPAR-CL, we calculated the transition frequencies of each of the four nucleotides, including the U–C transition rate at each genomic U position. For DMS-MaPseq, similar analysis was conducted but we focused on the overall mutation rates of A and C genomic positions. Between test groups and respective controls (4SU+/UV+ and 4SU+/UV- for vPAR-CL, DMS+ and DMS- for DMS-MaPseq), we compared the mutation/transition rate at the same genomic position, to yield the fold change map, as representations of vPAR-CL or DMS-MaPseq signals.

A background filter was applied to both vPAR-CL triplicates (Figure 4A–D) and DMS-MaPseq data (Figure 5D, E), to ensure reliable data analysis and to avoid potential false positives. For vPAR-CL, the background threshold is determined as the highest 5% of U–C transition rate in the uncrosslinked control group (4SU+,UV–). In the correspondent crosslinked group (4SU+,UV+), we removed any datapoint with U–C transition rate below this threshold, as it is indistinguishable from background fluctuation. An example of applying background threshold for vPAR-CL data can be found in Supplemental data 3. For DMS-MaPseq, similar background threshold was determined as the highest 5% of A/C mutation rate, in the DMS-untreated control group (DMS-). In DMS-treated group (DMS+), only the datapoints passing the background threshold were used to compile the fold change maps of mutation rate changes.

### RNA secondary structure prediction

RNA secondary structure prediction was conducted with *RNAstructure* (49) with 310.15 K temperature and maximum loop size = 30. ‘*Fold*’ (49,50) and ‘*Partition*’ (51) were used to predict the structure and base pairings within individual RNA and calculate the base pairing probability, respectively. The most significant DMS-MaPseq signal sites were applied as unpaired constraints in structure prediction. No other constraints applied to the rest genomic sites, regardless of the DMS-MaPseq signals. The predicted structure file was then re-organized and certain nucleotides were highlighted for graphical purposes with *StructureEditor* (v.1.0), provided by *RNAstructure* suite.

### Mutated virus with disrupted vPAR-CL sites

Plasmids containing FHV genomes were used as PCR templates. Universal upstream primer (TGCATAATTCTCTTACTGTCATGCCATCCGTAAG) and downstream primer (TAAGAGAATTATGCAGTGCTGCCATAACCATG) were used in combination with mutation primers (Table 1) to generate overlapped PCR fragments (Phusion High-Fidelity DNA Polymerase, NEB), with disrupted RNA structure at each selected vPAR-CL site. These overlapping fragments were then cloned into competent cells with standard In-Fusion HD Cloning (TaKaRa) techniques. The plasmids containing mutated FHV RNA 1 or RNA 2 sequences were then sanger-sequenced to confirm mutation.

**Table 1.** Candidate vPAR-CL sites for mutational assays. 11 consistent vPAR-CL sites for RNA 1 (within 10 RNA structures, as U1151 and U1910 are located on the same stem) and 5 sites for RNA 2 are listed, as well as their DMS-MaPseq predicted secondary structures, mutation primers and substituted nucleotides

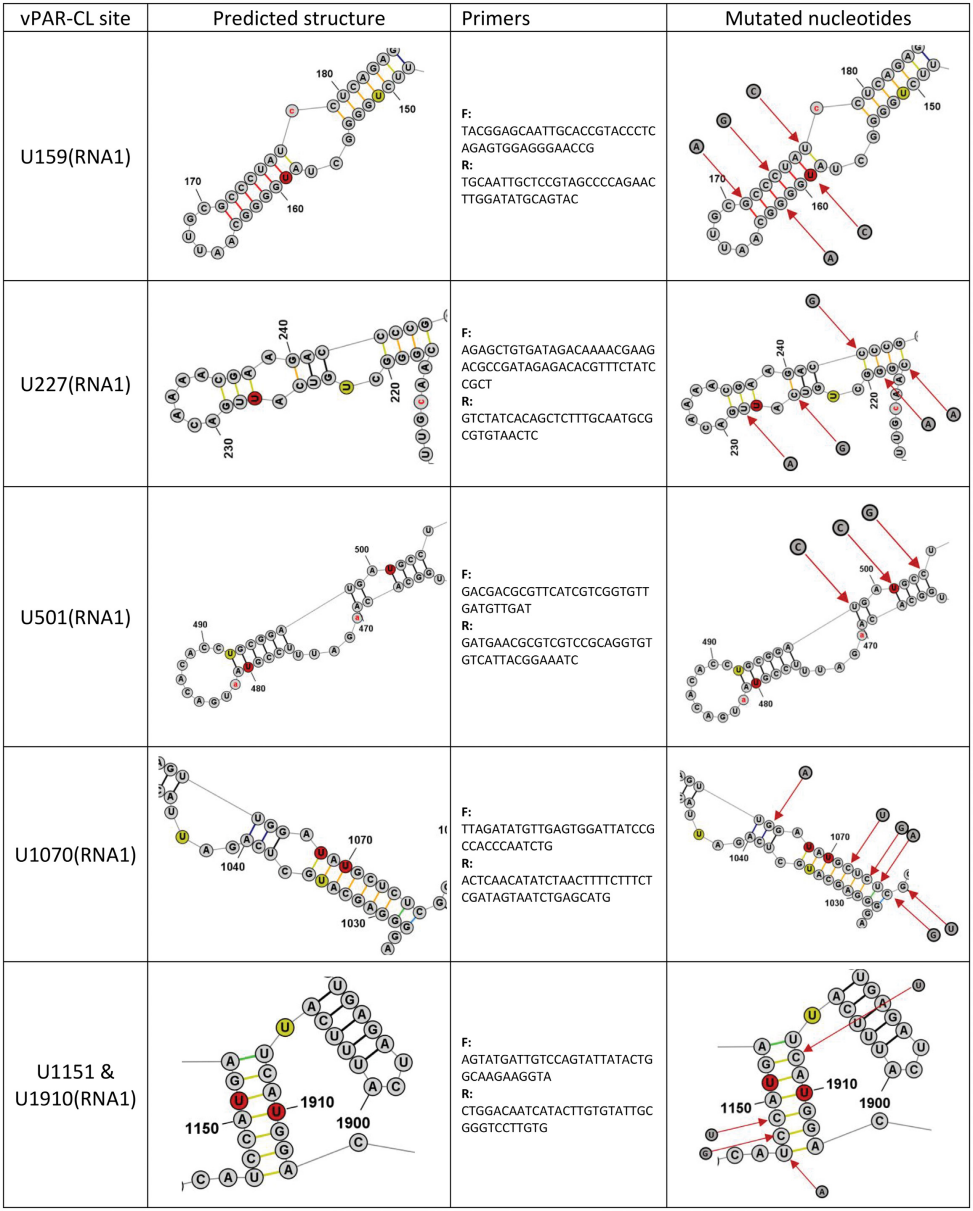 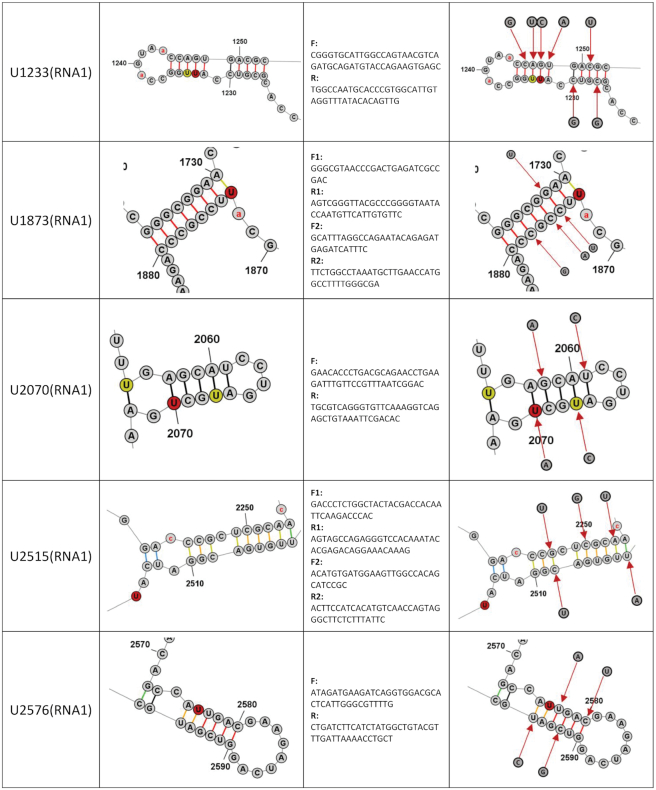 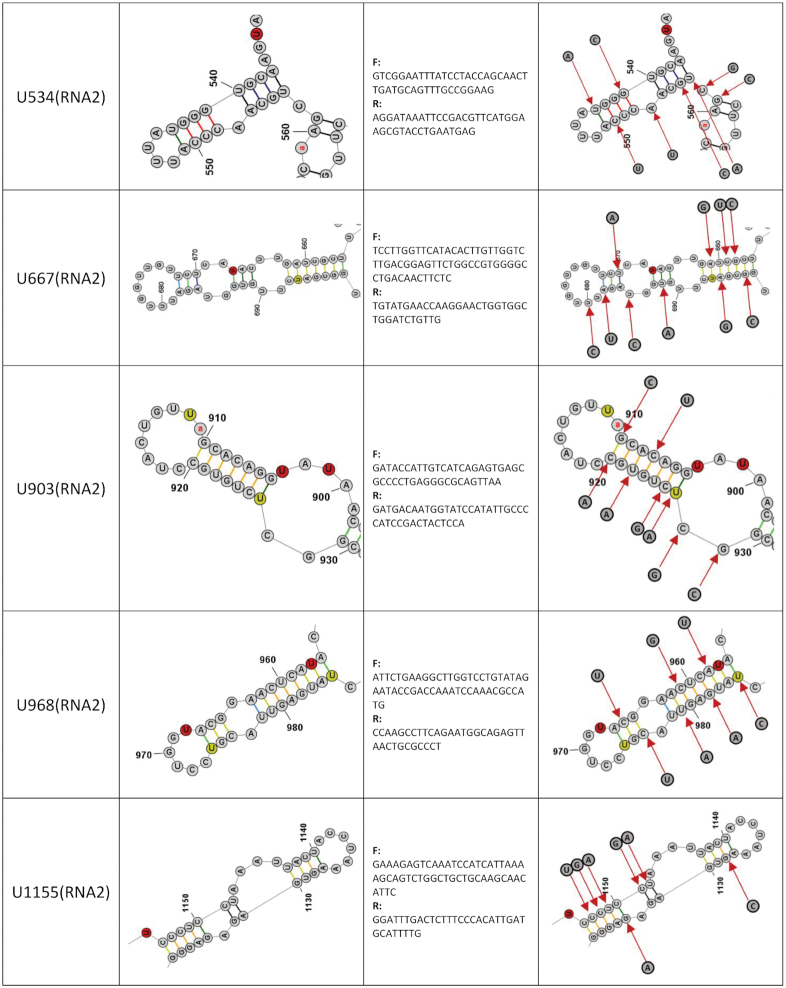 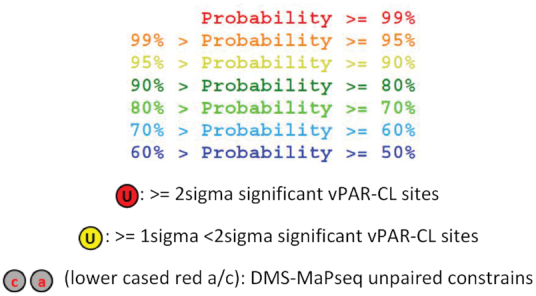

To generate mutant viruses, the plasmids containing vPAR-CL site mutations were used to transfect S2 cells with above-stated methods. Each mutant transfection consisted of equal amount of one mutated RNA genome with disrupted vPAR-CL site, and wt genome of the other RNA (Figure 7). These P0 mutant viruses were allowed to propagate in cell culture until 3 days post induction. Similar to before, P1 mutant viruses were generated by inoculating naïve S2 cells with P0 cell culture/virus mix.

### Relative virulence of mutant viruses

The virulence of P0 vPAR-CL mutant viruses was measured by transfecting S2 cells with plasmids containing mutant viral genomes. For this experiment, transfection was conducted in black 96-well plate, with each well seeded with 25k S2 cells. The transfection reagents and methods were similar to above, except for scaling down to adapt for 96-well plate. For each mutant virus, 100 ng plasmids of each mutant genome and the other wild type genome were used. alamarBlue was incubated with cell culture for 4 h, before detecting fluorescence with EnSpire plate reader (PerkinElmer) at 560 nm excitation and 590 nm emission. The relative fluorescence then normalized reverse-ratiometrically with mock transfection = 0% and FHV wt RNA transfection = 100%.

The relative virulence of P1 vPAR-CL mutant viruses was measured via infecting S2 cells with purified P1 mutant viruses. The P1 mutant viruses were purified through sucrose cushion (30% sucrose 10 mM Tris pH 7.4, ultracentrifuge 80k rpm for 1.5 h), PEG precipitation (4% v/v PEG8000), DNase I and RNase A treatment, buffer exchanged and concentrated with PES membrane protein concentrator (100K MWCO). The concentration of purified P1 viruses was confirmed by SDS-PAGE gel electrophoresis and densitometry. 25k S2 cells were seeded in black 96-well plate and 0.12 ng (approximately equivalent to MOI = 1 (37–39)) of serial diluted P1 viruses was used to infect each well. Standard alamarBlue assay was conducted as before, at 24 h post infection, to measure cell viability. The relative fluorescence then normalized reverse-ratiometrically with mock infection = 0% and purified wt FHV infection = 100%.

### RT-PCR

Transfected P0 vPAR-CL mutants were sampled for RT-PCR to detect RNA production. Total RNA was extracted from transfected cells and media with Direct-zol RNA kit (Zymo Research), and DNase I in-column digestion was conducted to remove plasmids. For each mutant and control, 200 ng of total RNA was used as template for RT-PCR. RT reactions was conducted with SuperScript III reverse transcriptase (Invitrogen), per manufacture's protocol. PCR was conducted with OneTaq^®^ (NEB), per manufacture's protocol. The entire RT-PCR reaction was analysed by agarose gel electrophoresis.

### SDS-PAGE and western blot

After collecting P0 transfections, the cell/virus/supernatant mix was centrifuged at 1000 × g for 10 min. Supernatant fraction was removed and collected thereafter. The cell pellet was washed once with 1× PBS and centrifuged as before. The washed cellular fraction was then resuspended in 1× PBS and 1× cOmplete (Roche). 150 μl of supernatant (of each sample) was supplemented with 1× cOmplete and then reduced with vacuum centrifuge prior to SDS-PAGE.

All SDS-PAGE assays were conducted with Bolt 4–12% Bis–Tris Plus Gels (Invitrogen). Membrane transfer was conducted with iBlot 2 Dry Blotting System (Invitrogen) with standard protocol. Western blot was conducted with iBind Western Device (Invitrogen) with standard protocol. Rabbit anti-FHV polyclonal antibody was given as a kind gift from Dr Vijay Reddy (Scripps Research), which was labelled with Alexa Fluor 488 goat anti-rabbit IgG (Invitrogen). Prior to membrane transfer, part of SDS-PAGE gel was cut and stained with Coomassie brilliant blue R-250 to highlight α-tubulin (55 kDa) as a loading control.

## RESULTS

### Viral photoactivatable-ribonucleoside-enhanced crosslinking (vPAR-CL)

**Figure 1.**
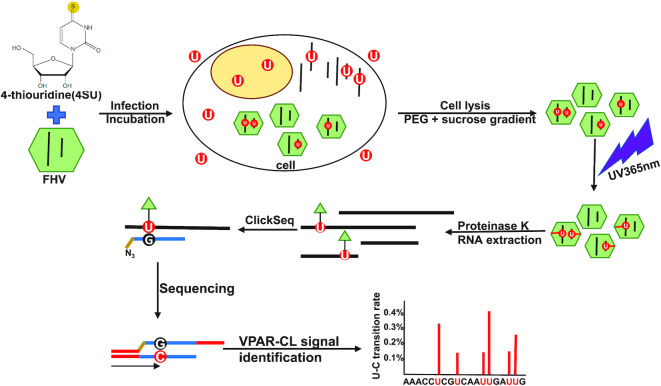
Viral photoactivatable-ribonucleoside-enhanced crosslinking (vPAR-CL) method allows for rapid and high-throughput detection of viral RNA–capsid interactions within virus particles. 4-Thiouridine (4SU) is supplemented to S2 cells during Flock House virus (FHV) infection. After incubation, purified viruses are irradiated with 365nm UV to induce RNA-protein crosslinks. After proteinase K digestion, crosslinked RNAs are purified and used as templates for RNAseq using ClickSeq. By mapping RNAseq reads to the viral genome, the crosslinked sites are characterized by elevated U to C transition rates.

PAR-CLIP (photoactivatable-ribonucleoside-enhanced crosslinking and immunoprecipitation) is a well-established method for identification of RNA–protein binding sites and can provide nucleotide-resolution through analysis of specific uridine to cytidine transitions that occur at the site of RNA-crosslinking during cDNA synthesis (33,40,52). Here, we simplified the technique by applying a similar approach to purified virions of an RNA virus, Flock House virus (FHV), thereby removing the necessity of immunoprecipitation, and hence deriving the name ‘vPAR-CL’. A schematic of the process is illustrated in Figure 1. In vPAR-CL, photoactivable nucleotide 4-thiouridine (4SU) was provided to cells in culture at the time of infection with FHV. 4SU is rapidly taken up and metabolized into 4-thiouridine triphosphate (4S-UTP) by cultured cells without significant cytotoxic effect (33,53). Upon infection, 4S-UTP is randomly incorporated into newly synthesized viral RNA, which is subsequently packaged into virus particles of FHV. Virus particles were isolated using established methods for virus purification by ultracentrifugation (20). Next, purified virus particles were subjected to UV 365 nm irradiation, yielding crosslinks between the thio-group of 4SU in the viral genomic RNA and amino acid residues in the protein capsid shell only if they are in close proximity (53,54). Virus particles were then disrupted by proteinase K treatment and a pool of viral RNAs with varied crosslinking sites was obtained. We then generated canonical random-primed next-generation RNA sequencing (RNAseq) libraries using ClickSeq (37,41,42) compatible with the Illumina HiSeq 1500 platform. Importantly, during reverse transcription, the crosslinked 4SU position results in a highly specific U-to-G mismatch during first strand cDNA synthesis (34–36). This mismatched G later is base-paired with C during PCR amplification. As a result, in the sequenced library, the emergence of C transitions at the reference U positions (U-C transitions) indicates crosslinking between capsid and 4SU sites.

**Figure 2.**
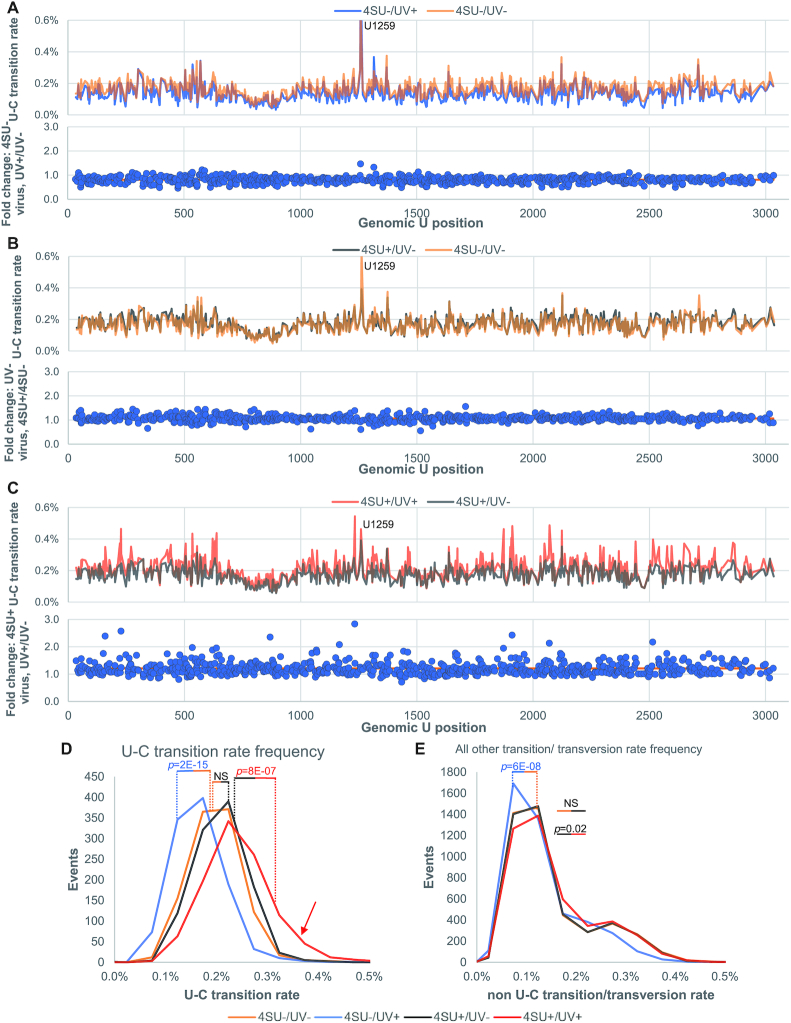
U-C transition rate elevation is specific to 4SU-induced crosslinking. Using FHV RNA 1 as an example, several control experiments were conducted to ensure specificity of vPAR-CL signals. (**A**) Effects of UV exposure to wt FHV (4SU-) were compared. We did not observe substantial U–C transition rate elevation. (**B**) Effects of 4SU incorporation were compared to FHV without UV exposure (UV–). We did not observe substantial U-C transition rate elevation. (**C**) We observed significantly increased U–C transition rates, only when 4SU-containing FHV was irradiated with UV (4SU+/UV+). (A–C) Upper panels illustrate the absolute U–C transition rate for each condition while the lower panels show the relative fold change between the two respective conditions. The orange line in fold change represents the average fold change in each experiment. A U1259C minority variant resulting in an apparent U–C mutation is indicated. (**D**) The distribution of U–C transition frequencies: only when both 4SU and UV are present (4SU+/UV+, red line), did we observe a significantly higher U–C transition rate (red arrow). **(E)** While 4SU-/UV+ resulted in a reduced transition rate, no significant difference was found in other samples (all A,G,C-transitions and transversions, or U–A/U–G transversions). (D and E) Statistical assays were conducted with Two-Sampled Kolmogorov–Smirnov (K–S) test with α = 0.05. NS = not significant.

**Figure 3.**
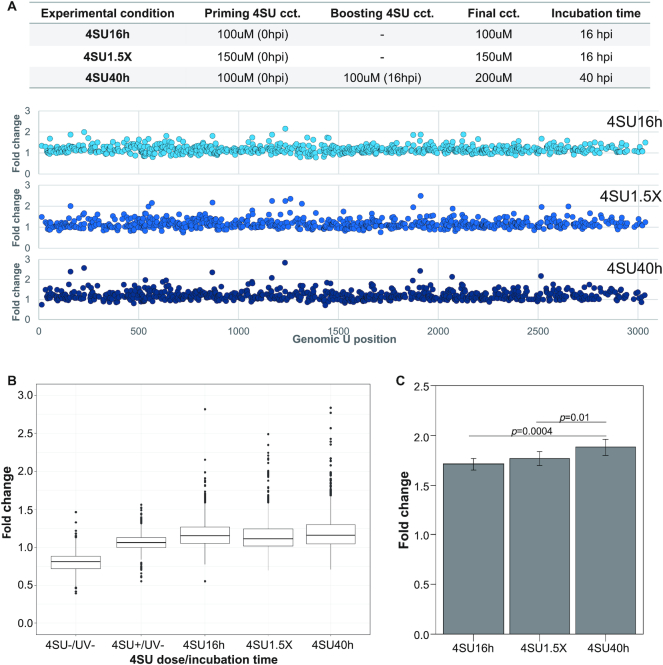
vPAR-CL signal intensities increase with higher 4SU dose and longer incubation time. Using FHV RNA 1 as an example, (**A**) three experimental conditions were tested for impact to vPAR-CL signals. In each experiment, the priming dose of 4SU was supplemented to cells at the time (0 hpi) of FHV infection (MOI = 1). We observed that the intensities of vPAR-CL signals (i.e. fold change of U-C transition rate between 4SU+/UV+ and 4SU+/UV–) were impacted by 4SU concentration and time of incubation as certain genomic U positions showed higher signals with higher concentration and longer incubation time. We observed good correlation coefficient of vPAR-CL signals under different conditions, indicating reproducibility (Supplemental Figure S3). (**B**) With or without crosslinking, the average vPAR-CL signals among all 4SU-containing FHVs is similar. However, the outliers of crosslinking groups showed higher signals than control (4SU+/UV–), and the magnitude of outlier signals correlated with 4SU concentration and incubation time. This indicated that 4SU-induced crosslinking only affected certain specific positions on FHV genome. (Whiskers are defined as 1.5× interquartile range (IQR) in all boxplots (Tukey boxplot)). (**C**) We sampled top 5% vPAR-CL signals from three experiment groups and determined that optimal vPAR-CL signal was achieved under 4SU40h condition, which was therefore applied to all further vPAR-CL experiments. Statistical assays were conducted with standard *t*-test (two-tailed).

To validate the vPAR-CL methodology, we first sought to determine if there was a substantial increase in U–C transition rate as a specific consequence of 4SU-capsid crosslinking. We performed a series of experiments in which wild-type (wt) FHV without 4SU (4SU-) or 4SU-containing FHV (4SU+) was treated with (UV+) or without (UV–) UV irradiation. As illustrated for FHV RNA 1 in Figure 2A, we plot the measured U-to-C (U–C) transition rate across the genome and calculate the fold change at each U position. A small number of positions, such as nt. U1259 on RNA 1, showed significantly higher U–C mutation rates than average under both conditions (standard independent two tailed *t*-test with *P* = 2.82E–53). This possibly reflects a minority variant present during virus passaging. Other than this, UV irradiation alone was not sufficient to increase U–C transition rate in the absence of 4SU (4SU–/UV+). In contrast, 4SU–/UV+ exhibited lowered U-C transition rate, for an unknown reason (Figures 2D and 3B). Similarly, we measured the influence of 4SU substitutions in FHV genomic RNA without UV exposure (4SU+/UV–) (Figure 2b). We also did not notice an increase in the U–C transition rate (Figure 2D). We only observed increased U-C transitions when 4SU and UV irradiation both were present (4SU+/UV+, *P* = 8E–07) (Figure 2C, D). This confirms the elevated U–C transition rate is indeed a specific result of 4SU-induced crosslinking. The FHV RNA 2 data of these experiments are shown in Supplemental Figure S1A. We also compared 4SU+/UV+ and 4SU–/UV+ viruses (Supplemental Figure S1B) and noticed that this comparison resulted in consistent yet even more substantial U–C transition fold change. This is likely due to the reduced transition rate in 4SU–/UV+ virus, as demonstrated in Figure 2D.

Histograms of the U-C transition rate frequencies at all genomic U positions are shown in Figure 2D. This demonstrates that under 4SU+/UV+ condition, more U positions exhibited high U–C transition rate (≥0.3%) than controls (4SU–/UV– and 4SU+/UV–). We also sought to determine if 4SU incorporation and UV exposure would induce any non-specific (non-U–C) transitions. A histogram of the frequencies of all non-U–C transitions/ transversions (A,C,G transitions/ transversions and U–A, U–G transversions) over all genomic positions is shown in Figure 2E. Importantly, 4SU+/UV+ FHV did not show any significant change in non-U–C transitions/transversions. We therefore conclude that the increased U–C transition rate is specific to 4SU-induced crosslinking.

### Magnitude of the vPAR-CL signal is associated with 4SU dose and incubation time

Our vPAR-CL method requires no immunoprecipitation to recover and enrich for crosslinked RNAs. However, this permits wild type uridine and/or uncrosslinked 4-thiouridine to persist in the RNA pool which may dilute the vPAR-CL signal. To investigate the conditions for achieving higher vPAR-CL signals, we conducted three parallel experiments (Figure 3). S2 cells were infected with FHV and incubated with 4SU at 100 μM (4SU16h) or 150μM (4SU1.5X). Viruses were harvested from infected cells 16 h post-infection. In a third experiment, we extended the incubation time to 40 h and applied 4SU in a ‘prime-boost’ manner, to reach a final concentration of 200 μM (4SU40h). Time-points were limited to 40 hours in order to avoid major cytopathic effect, usually seen at 48 h, and to minimize secondary rounds of FHV infection where virus particles may begin to release their genetic cargo. In each experiment, the U–C transition profile of 4SU+/UV+ virus was compared with corresponding 4SU+/UV– virus to yield vPAR-CL signals (fold change of U–C transitions). Results for FHV RNA 1 are shown in Figure 3A and RNA 2 in Supplemental Figure S2. We noticed that the concentration of 4SU and the incubation time of FHV/4SU had an impact on vPAR-CL signal intensities over a number of genomic U positions.

The same results were observed when we plotted the frequency of vPAR-CL signals for these three experiments, as well as two controls (Figure 3B). This shows that while the mean vPAR-CL signals and the distribution under all three conditions (4SU16h, 4SU1.5X and 4SU40h) and a 4SU+/UV– control were all comparable, the magnitude of outliers showed correlation to experimental conditions (4SU40h > 4SU1.5X > 4SU16h). This indicates that only certain 4SU substitution sites were available for crosslinking and therefore sensitive to the varied 4SU concentrations/incubation times. Again, for an unknown reason, the 4SU–, UV+ control (Figure 3B) showed a slightly lesser than 1-fold change vPAR-CL signal. Importantly, this does not interfere with our interpretation of vPAR-CL signals in crosslinked samples.

We noticed significant difference of vPAR-CL signal distribution between 4SU40h/4SU1.5X and 4SU1.5X and 4SU16h (Supplemental Figure S2B). To further assess whether experimental conditions impacted intensity of vPAR-CL signals at certain sites, we sampled the top 5% of vPAR-CL signals in each conditioned experiment (Figure 3C), as these signals most likely represent crosslinking sites that are sensitive to vPAR-CL experimental conditions. We noticed that, the 4SU40h group showed significantly higher vPAR-CL signals than the rest. For this reason, the 4SU40h experimental condition was applied to all further vPAR-CL experiments, unless otherwise mentioned. Despite the varied vPAR-CL signal intensities under different experimental conditions, the vPAR-CL signals presented good Pearson's correlation coefficient (≥0.6) between these parallel experiments (Supplemental Figure S3), which indicates high reproducibility of vPAR-CL experiments.

### Consistent vPAR-CL signals indicate structural tropism of encapsidated viral RNAs

**Figure 4.**
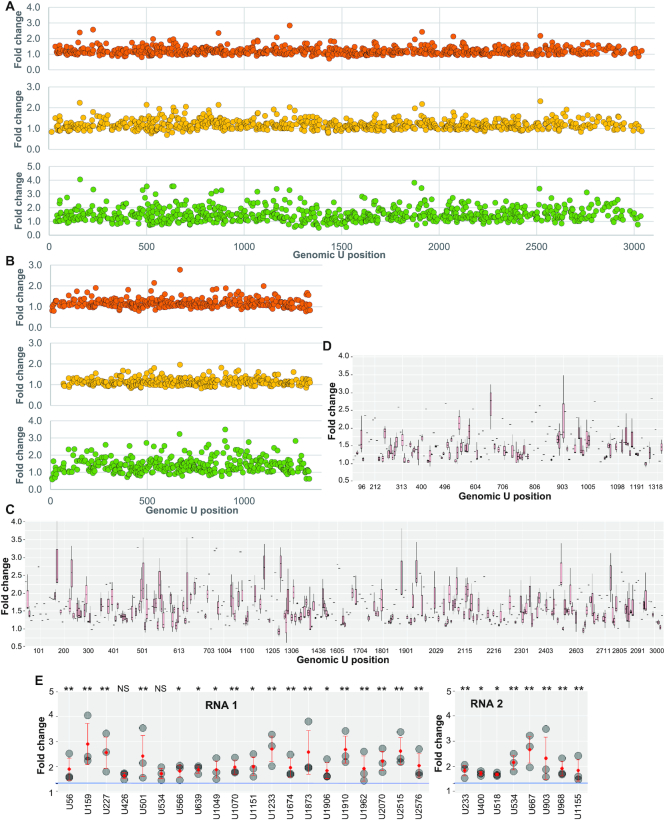
Consistent vPAR-CL sites revealed clear RNA tropism in FHV virion. Biological triplicate FHV vPAR-CL experiments, with signals plotted for (**A**) RNA 1 and (**B**) RNA 2. Biological triplicates’ vPAR-CL signals were box-plotted for (**C**) RNA 1 and (**D**) RNA 2. Signals below background threshold were removed, which resulted in vacant or singular signals over certain genomic positions. A number of sites on both RNA 1 and RNA 2 retained substantial signals in all triplicates, indicating replicable crosslinking sites between RNA and capsid. These consistent vPAR-CL sites also suggest a conserved tropism of FHV RNA cage inside virion. X-axis is not continuous. (**E**) Among these consistent vPAR-CL sites, most of them showed significantly higher vPAR-CL signals than the average. Grey circles: vPAR-CL signals of each biological replicate; red dots: mean signals. Blue line: average vPAR-CL signal of RNA 1 and RNA 2, respectively. *P < 0.05; **P < 0.01; NS = not significant. Statistical assays were conducted with standard *t*-test (two-tailed).

We applied the 4SU40h condition in three parallel experiments: three separate S2 cell cultures were incubated with virus and 4SU, and individually purified viruses were exposed to UV and thereafter proceeded to sequencing. As before, vPAR-CL experiments were conducted in pairs, with each vPAR-CL dataset comprising one crosslinked sample (4SU+/UV+) and one control with the same RNA but without UV irradiation (4SU+/UV–). To ensure reliable transition rate calculation, we selected for U positions with coverage of at least 10000 reads (an example of coverage map can be found in Supplemental Figure S4). This allowed us to detect reliable transition profiles over U34–U3034 on RNA 1, and U9–U1337 on RNA 2. The vPAR-CL signals of these three experiments were compared on each U position on viral RNA genomes (Figure 4A, B). We observed good Pearson's correlation coefficient (≥0.6) between these replicates (Supplemental Figure S5). To validate the consistent vPAR-CL signals, the signals over every U position were box-plotted over the triplicates (Supplemental Figure S6). This allows us to readily measure the mean signal strength and signal variation over the triplicates. In order to distinguish reliable crosslinking sites and avoid potential false positives, we removed any vPAR-CL signals in crosslinked sample by applying a conservative background threshold filter, which represents the highest 5% of vPAR-CL signals in the uncrosslinked control sample. (Figure 4C, D, Supplemental data 3). Among the most consistent vPAR-CL sites (passed background threshold in all replicates), we selected 20 sites in RNA 1 and eight sites in RNA 2 that showed the highest average vPAR-CL signals (Figure 4E, Supplemental data 3). *t*-test revealed most of these sites showed significantly (*P* < 0.05) higher vPAR-CL signals than average. As these same sites consistently displayed significant vPAR-CL signals over parallel biological replicates, this indicates a set of consistent RNA–capsid interactions in FHV virions, which further indicates a structural tropism of FHV RNA in association with the topology of virus capsid shell.

### Probing FHV RNA secondary structures in virions with DMS-MaPseq

Sequence conservation assays revealed that the most significant and consistent vPAR-CL sites showed conservation among common *alphanodaviruses* (Supplemental Figure S7). We sought to determine whether there is any sequence motif among the vPAR-CL sites. Significant (>2σ) vPAR-CL sites (28 sites from RNA 1 and 15 from RNA 2, Supplemental Figure S8) and their flanking sequences were analyzed with Discriminative Regular Expression Motif Elicitation (DREME, (55)) for possible sequence motif identification. However, no common motif was identified. This led us to hypothesize that the mechanism of RNA recognition by FHV capsids may be related to RNA structure rather than primary sequence. To reliably characterize the RNA secondary structures of vPAR-CL signals, we sought to experimentally determine the secondary structure of FHV RNA in virions.

**Figure 5.**
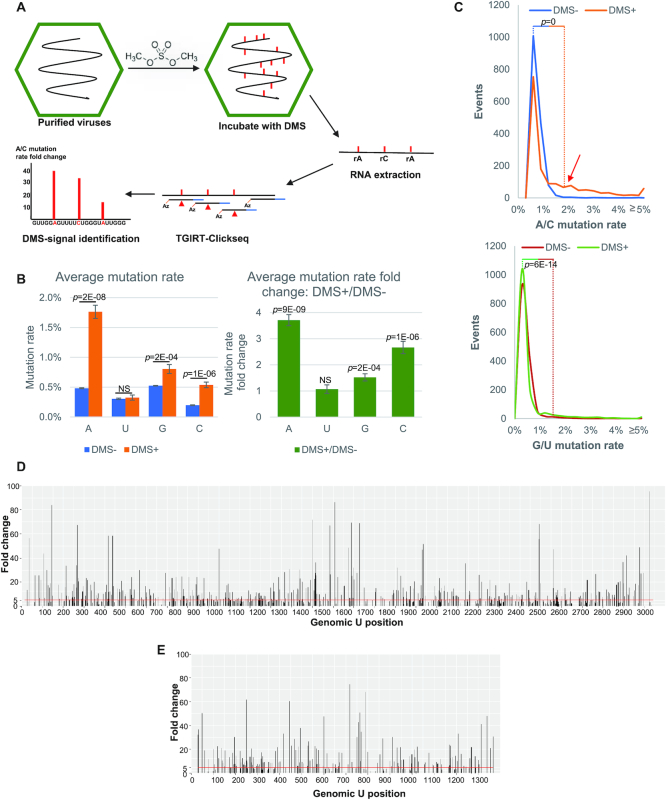
FHV DMS-MaPseq revealed ssRNA constrains in virions. (**A**) To predict the secondary structure of FHV RNA, we used DMS (dimethyl sulfide) to induce in virion methylation of unpaired adenines and cytosines. The extracted RNAs were subjected to ClickSeq library preparation with TGIRT^TM^-III enzyme, which generates mutations over methylated bases. DMS-MaPseq signal is represented by the mutation rate fold change over A/C positions. Red markers on RNA represent methylated ribonucleotides; red triangles on cDNA represent DMS-induced mutations. (**B**) Increased rates of A/C mutations were detected as a result of DMS treatment. Statistical assays were conducted with standard t-Test (two-tailed) with α = 0.05, NS = not significant. (**C**) DMS-treated virus exhibited significantly higher mutation rates over A/C positions (red arrow). Statistical assays are conducted with Two-Sampled Kolmogorov-Smirnov (K-S) test with α = 0.05. DMS-MaPseq profiles for (**D**) RNA 1 and (**E**) RNA 2, respectively. Background noise was removed. Red line represents the average DMS-MaPseq signal.

Dimethyl sulfate mutational profiling with sequencing (DMS-MaPseq) provides a reliable and high-throughput method to probe RNA secondary structures *in vivo* (43,44,56). The resulting constraints provide improvements to thermodynamic mapping and free energy-based secondary structure prediction. We performed DMS-MaPseq using the TGIRT™-III enzyme but in combination with ClickSeq to generate RNAseq libraries (‘TGIRT-ClickSeq’) (Figure 5A), demonstrating that the TGIRT™-III enzyme is compatible with ClickSeq. DMS-MaPseq induces RNA modifications to unpaired adenines and cytosines (and guanine to a lesser level (57)) across the viral genome. Therefore, as expected, in comparison to untreated control virus (DMS–), DMS-treated FHV (DMS+) has a higher average mutation rate over genomic A/C positions (Figure 5B). We plotted the frequency of mutation rates over A/C or G/U positions and noticed a significantly higher mutation rate frequency over A/C positions (Figure 5C). We analyzed A or C positions with at least 10k read coverage, which correspond to nt. 14−3043 on RNA 1 and nt. 11–1378 on RNA 2. Similar to vPAR-CL data, DMS-MaPseq signal represents the mutation rate fold change between DMS-treated virus (DMS+) and untreated control virus (DMS-), at all genomic A or C positions. Likewise, we removed potential false-positive signals by applying a background noise threshold, retaining only the genomic sites with mutation rate higher than this threshold. The resulting DMS-MaPseq profile of FHV (Figure 5D and E) showed clear signals up to 100-fold change over both RNA 1 and 2. The un-refined DMS-MaPseq profiles with background noise, and mutation rate comparison between DMS-treated and untreated viruses are shown in Supplemental Figure S9.

### vPAR-CL sites favored double stranded RNA structures and were highly clustered

**Figure 6.**
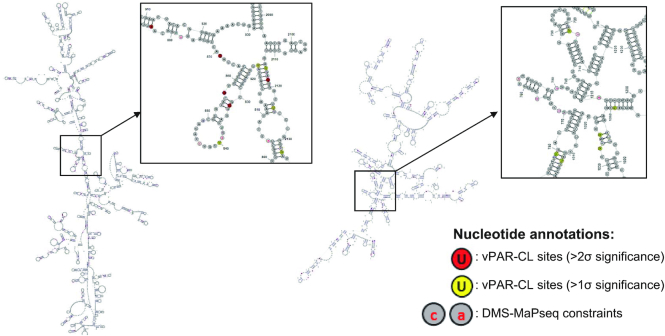
FHV RNA secondary structures in virions revealed vPAR-CL sites are highly clustered and enriched in dsRNA regions. FHV RNA in virion secondary structures were predicted with DMS-MaPseq-imposed constrains. 58% of RNA 1 and 60% of RNA 2 is double-stranded. Snapshots of RNA 1 (left) and RNA 2 (right) are shown. Full scaled maps can be found in Supplemental data 1 and 2. vPAR-CL signal sites of different significance were color annotated. The highly clustered nature of vPAR-CL sites can be revealed both locally and remotely, when RNA secondary structures are taken into account (insets).

We incorporated the DMS-MaPseq data into free energy based thermodynamic prediction, by introducing a series of ‘soft’ constraints. Only the most significant (>2σ) DMS-MaPseq sites (60 sites in RNA 1 and 30 sites in RNA 2) were imposed as unpaired constraints in the *RNAstructure Web Server* (49) with ‘*Fold*’ algorithm (49,50). Regardless of their DMS-MaPseq signals, the remaining genome positions were left without any constraints, to allow maximum prediction flexibility. Importantly, predictions were only conducted within the same RNA molecule (RNA 1 or RNA 2), omitting any potential intra-molecule pairings. We thereby constructed a DMS-MaPseq-imposed secondary structure map of complete FHV RNA genome (snapshots in Figure 6, full-scaled maps of RNA 1 and RNA 2 are also provided in Supplemental data 1 and 2). Despite the low number of introduced constraints, we were able to substantially adjust the thermodynamic structure of FHV RNAs. With the 60 RNA 1 constraints, 37% (1145/3107) of nucleotides underwent refolding compared to the unconstrained model, yielding different paired/unpaired patterns. Similarly, with the 30 RNA 2 constraints, 20% (273/1400) nucleotides underwent refolding. The dot-bracket maps comparing the differences between unconstrained and constrained folding can be found in Supplemental Figure S10. Together, our DMS-MaPseq-improved RNA in virion structure revealed that 58% of RNA 1 and 60% of RNA 2 are double-stranded.

In combination with vPAR-CL data, we noticed that the significant RNA–capsid interaction sites favored double-stranded base-pairing. In RNA 1, among the 28 most significant (>2σ) vPAR-CL sites, 22 are located in double-stranded regions (*χ*^2^ = 4.7, *P* = 0.03, chi-square test), whereas three sites were 1 nt. adjacent to double-stranded stems. In the much shorter RNA 2, 8/15 of most significant vPAR-CL sites are located in dsRNA stems (*χ*^2^ = 0.3, *P* = 0.58, chi-square test), whereas 3 are 1 nt. adjacent. The lower frequency of RNA 2 vPAR-CL sites found in double-stranded regions of RNA is expected as RNA 2 represents a shorter RNA with fewer vPAR-CL sites. However, these data suggest that RNA 1-capsid interactions may predominate in the dodecahedral dsRNA cage observed immediately inside FHV particles. In Table 1, we illustrate the detailed structures of 16 vPAR-CL sites (11 on RNA 1 and 5 on RNA 2) that presented with highest consistency and average vPAR-CL signals (Figure 4A–D).

We also noticed that the distribution of vPAR-CL signals was uneven and highly clustered. Numerous vPAR-CL stems showed more than one vPAR-CL sites with at least >1σ significance (Supplemental data 1, 2, some examples were listed in Table 1). The clustered nature of certain vPAR-CL sites can only be appreciated by considering RNA secondary structure (as illustrated in Figure 6). We calculated the average shortest distance between adjacent vPAR-CL sites. On RNA 1 (3107 nt.), from 721 uridine sites, 102 showed >1σ significant vPAR-CL signal. The average shortest distance between these vPAR-CL sites is 7.4 nucleotides, which is substantially shorter than the average shortest distance of 102 random uridines (30.47 nucleotides). On RNA 2 (1400 nt genome with 351 uridines), the average shortest distance among 45 vPAR-CL sites (>1σ significance) was 8.8 nt, which is also shorter than the average shortest distance of 45 random uridines (31.11 nt).

Notably, by combining vPAR-CL data and DMS-MaPseq-imposed RNA structure, we are able to characterize a stem loop site which is structurally near identical to a previously predicted stem loop (nt. 168–249) on RNA 2 (31) (Supplemental Figure S11). This stem loop site, as well as the flanking sequences (nt. 210–249) has been determined to be essential for RNA 2 encapsidation. We identified three vPAR-CL signals within this region, consistent with role of this stem loop site in RNA 2 packaging.

### Structurally-disrupted vPAR-CL sites impact FHV lifecycle and fitness

**Figure 7.**
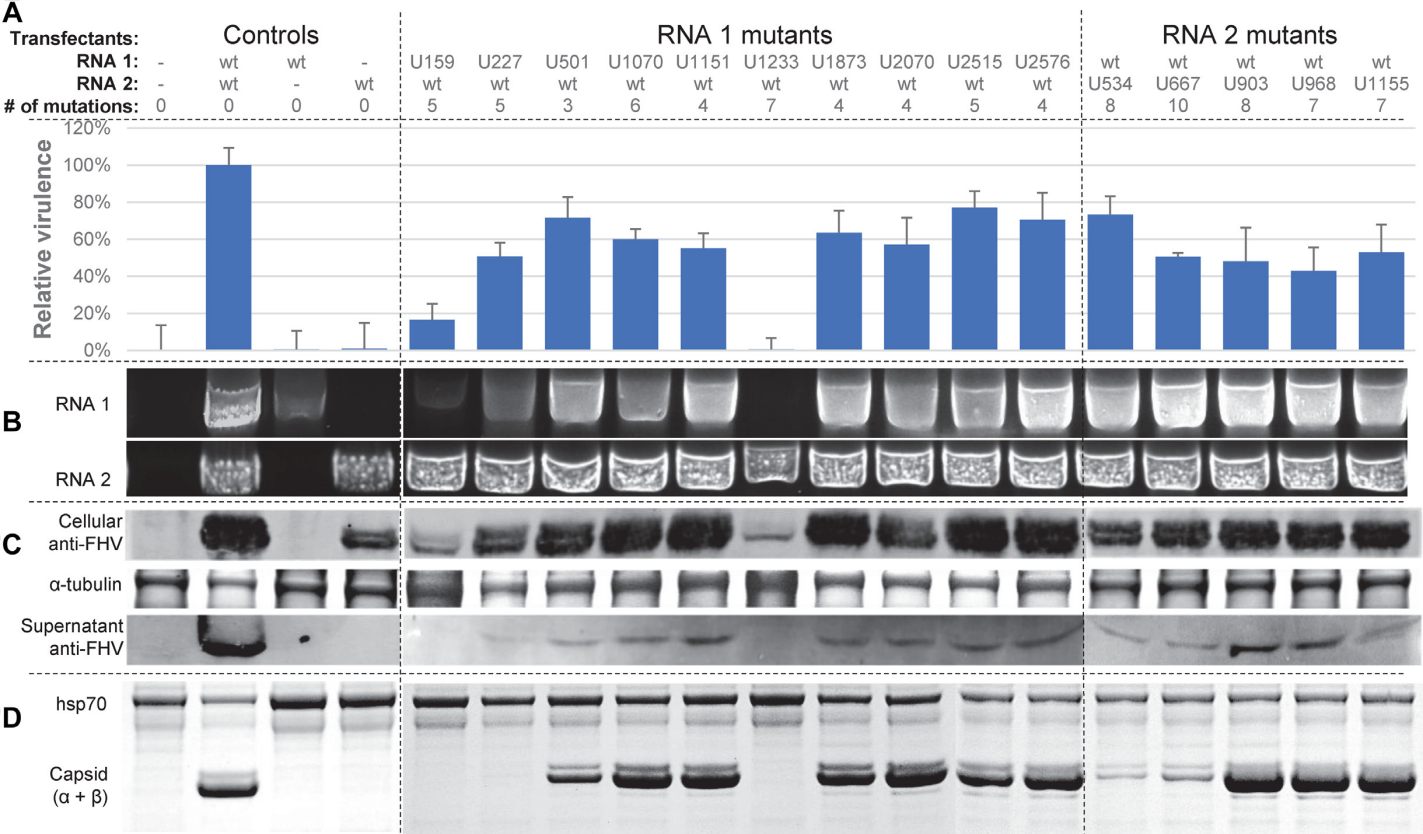
Synonymously re-coded vPAR-CL sites impact FHV lifecycle and fitness. Plasmids containing FHV RNA 1 or RNA 2 with mutated vPAR-CL structures were used to transfect S2 cells to yield P0 mutant viruses. For the mutants, the RNA 1 or RNA 2 transfectant (e.g. U159) refers to the vPAR-CL sites listed in Table 1. (**A**) Relative virulence of P0 mutant viruses was determined with alamarBlue assay to measure cell viability after transfection. (**B**) 200 ng of total cellular RNA from each transfection was analyzed by RT-PCR to measure the accumulation of FHV RNAs. (**C**) Cellular and supernatant FHV capsid production was detected with anti-FHV capsid antibodies. Coomassie-stained α-tubulin is shown as loading control. (**D**) P1 viruses were purified and filtered with 100K molecular weight filter. Mutant virus production was verified with SDS-PAGE gel. Heat shock protein 70 (hsp70) provides a loading control.

To determine whether the identified vPAR-CL sites have a biological function, we selected 11 vPAR-CL sites from RNA 1 and 5 vPAR-CL sites from RNA 2 as candidate sites (Table 1). Referring to the DMS-MaPseq-corrected FHV RNA structure maps (Figure 6), we introduced synonymous mutations to disrupt the double-stranded RNA regions of the vPAR-CL sites (or the nearest stem of certain vPAR-CL sites, i.e. U2515 on RNA1, U534, U903, U968, and U1155 on RNA2). The predicted structure of these vPAR-CL sites, primers and synonymously-mutated nucleotides are listed in Table 1. Plasmids containing these point mutations were transfected into S2 cells. Each transfection consisted of either a mutated RNA 1 and wild-type RNA 2, or a mutated RNA 2 and wild-type RNA1 (Figure 7). After transfection, induction and incubation, cell viability of each transfected mutant was determined with alamarBlue assay (Figure 7A). Almost all mutant virus transfections (P0 virus) showed reduced cytopathic effect compared to transfection with wild-type FHV RNAs. Notably RNA 1 mutants U159, and U1233 resulted in little to no detrimental effect to S2 cells.

**Figure 8.**
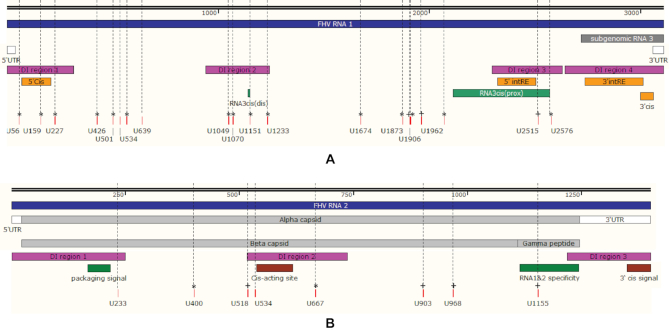
vPAR-CL signals coincide with known motifs of FHV. (**A**) On RNA 1, four regions that are highly conserved/retained in defective-interfering RNAs (DI regions 1–4) (37) are shown in pink; four RNA 1 replication regulatory elements are shown in orange (5′Cis (58), 5′intRE (59), 3′intRE (59) and 3′Cis (75)); two RNA 3 regulatory elements and a putative RNA 3 subgenomic promoter region are shown in green (RNA3cis(dis) (59), RNA3cis(prox) (59)); the candidate vPAR-CL sites (listed in Figure 4E) are shown as red bars on bottom. * denotes double-stranded vPAR-CL sites; + denotes single-stranded vPAR-CL sites that are 1 nt. adjacent to dsRNAs. (**B**) On RNA 2, three regions that are highly conserved/retained in defective-interfering RNAs are shown in pink (DI regions 1–3 (37)); a mid-genome *cis*-acting replicational element (71) and a 3′ *cis*-acting regulatory element (75) are shown in brown; a RNA 2 packaging signal (31) and a capsid site essential for RNA 1&2 specificity (25) are shown in green.

Total cellular RNA was extracted from transfected cells and in-column DNase digestion was conducted to remove remaining plasmids. From each transfection, 200ng of purified RNA was used as template for RT-PCR to detect FHV RNA (Figure 7B, original gel images in Supplemental Figure S12). We noticed that accumulation of FHV RNA 2 was unaffected by any vPAR-CL mutants, while RNA 1 accumulation varied drastically among RNA 1 mutants. Notably, RNA 1 mutant U1233 yielded undetectable levels of RNA 1, while RNA 2 production was less affected. RNA 1 mutant U159 also produced marginal amount of RNA 1, and U227 produced substantially less RNA 1 than that of control or RNA 2 mutants. The replication deficiency of these three mutants agreed with our findings of their low virulence (Figure 7A). Interestingly, these three sites are found within or adjacent to previously described FHV RNA regulatory regions (58,59) (Figure 8). The importance of these three sites in both RNA–capsid interaction shown here and RNA replication regulation indicates that the same motifs in the RNA genome are involved in multiple stages of the viral life-cycle, consistent with the notion that replication and RNA genome packaging are tightly coupled processes (27,28).

To confirm capsid production, we separated cells and supernatant from the transfected cells. Western blots with anti-FHV were used to detect capsid proteins in both cellular components and supernatants (Figure 7C). In the cellular fraction, we readily detected both mature (α-peptide) and autoproteolytically cleaved capsid protein (β-peptide) in all mutants. However, reduced capsid yields were found in U159 and U1233 mutants, possibly due to the observed RNA 1 replication deficiency. In supernatant fractions, the U159 and U1233 mutants resulted in undetectable level of capsid protein, while U227 resulted in detectable but very marginal amount of capsid production. This confirmed that the mutations at these three vPAR-CL sites have significant impact on virus production in S2 cells.

To expand mutant viruses, we further inoculated naïve S2 cells with equal amount of transfected P0 cell mix. From the inoculated P1 cell culture, we observed different degrees of cytopathic effect (CPE) under microscope (Supplemental Figure S13), which was in agreement with earlier findings. P1 mutant viruses were nuclease treated, PEG precipitated, and purified with PES membrane protein concentrator to remove potentially unassembled capsid subunits. The presence of virus particles was confirmed with SDS-PAGE (Figure 7D), and virus yield was calculated by densitometry. Similar to before, we failed to detect virus production of U159 and U1233 mutants, while U227 mutant resulted a marginal virus production which can only be detected by western blot but not with SDS-PAGE. This result also agreed with our western blot analysis (Figure 7C).

We further tested P1 mutant virus relative virulence by infecting cells with mutants at MOI = 1 (Supplemental Figure S14). Most mutant viruses still resulted in varied but inferior virulence, in comparison to wild type virus.

## DISCUSSION

### vPAR-CL methodology

Photoactivatable nucleoside analogs were successfully utilized in the past to enhance crosslinking efficiency and hence, providing approaches to study RNA–RNA and RNA-protein interactions (reviewed in (60)). Thionucleobases such as 4-thiouridine (4SU) and 6-thioguanosine (6SG)) allow for highly efficient crosslinking at 330–365 nm excitation spectrum (61), as well as minimum RNA structure perturbation (60,62), lower cytotoxicity (33,53,54,63), and reduced photochemistry and/or photodamage (60,62). Importantly, the 4SU/6SG incorporated RNA can lead to specific base mismatches during reverse transcription (U–C, and G–A)(34–36), which enables high-throughput screening as indications of crosslinking. This is best illustrated with PAR-CLIP (33), which pin-points crosslinking sites with nucleotide resolution. PAR-CLIP has been successfully applied in the past to identify crosslinking sites of Argonaute 2, embryonic lethal abnormal vision (ELAV) protein, pumilio homologue 2 (PUM2), and insulin-like growth factor proteins (33,64). Utilizing the same crosslinking rationale and oligo(dT) magnetic beads capture, a reverse application unbiasedly depicted the RNA-binding protein (RBP) profiles in HeLa cells (65).

As its primary purpose, PAR-CLIP was designed to screen the entire transcriptome for RNA sequences binding to an RBP of interest. Typically (33,40,52), PAR-CLIP is conducted by incubating cell cultures with 4SU, followed by 365 nm UVA irradiation, cell lysis, RNase T1 digestion, immunoprecipitation of RBP, second RNase T1 digestion, de-phosphorylation, radiolabeling, SDS-PAGE and electro-elution, proteinase K digestion, and RNA extraction. The recovered crosslinked RNA then is used as a template for cDNA library preparation and deep sequencing. A natural prerequisite is large amounts of starting materials (between 10^8^ and 10^9^ cells (40)).

The unique aspect of our simplified vPAR-CL method is that we applied the similar PAR-CLIP principles to an RNA virus (FHV), which can be easily separated from cellular components. Crosslinks within purified virus particles allow us to: (i) eliminate the need for immunoprecipitation to recover crosslinked RNA; (ii) look for specific in virion interactions between viral RNA genomes and viral capsid proteins; (iii) study a reductionist and highly controlled microenvironment. The greatly simplified vPAR-CL methodology, in combination with ClickSeq library construction technology (42), granted the ability to conduct an experiment with as little as 2 μg of purified FHV particles. A single T25 flask of S2 cells can generate ample amounts of pure 4SU-containing viruses to conduct multiple vPAR-CL experiments.

In our vPAR-CL method, the final pool of purified viral RNA can contain large number of canonical uridines, or uncrosslinked 4SUs. As a consequence, the signal of any randomly generated, non-specific crosslinking events will be largely diluted into background level. Only the consistent crosslinking sites due to homogeneity in RNA–capsid interactions within a viral population can readily provide distinguishable vPAR-CL signals from background. Therefore, in contrast to the canonical PAR-CLIP approach where only crosslinked RNA fragments are sequenced, we are also able to identify regions of the viral genome where there is no reproducible vPAR-CL signal, either due to a lack of RNA–capsid interactions or heterogeneous interactions. This is best illustrated in Figure 3B, where the background noise levels are largely unchanged, with or without crosslinking. We speculate that these regions may correspond to RNA present in the internal cavity of virus particles rather than comprising part of the decahedral RNA cage.

In both PAR-CLIP and vPAR-CL, there are intrinsic limitations of 4SU-induced crosslinking. Firstly, crosslinking is only limited to U positions. Any potential interaction between protein and other nucleotides is undiscoverable. Next, 4SU crosslinking with protein is affected by reactivity of amino acid side chains (34,36), with aromatic amino acids (phenylalanine, tyrosine, and tryptophan) being predominant targets but also lysine and cysteine (34). Consequentially, not all RNA–protein interactions can be depicted by vPAR-CL or PAR-CLIP, and certain interactions may not result in crosslinking.

### FHV vPAR-CL experiments and data analysis

Several approaches were used to ensure reliable interpretation of vPAR-CL signals on FHV: (i) to ensure reliable interpretation of transition rate, we limited our analysis to genomic positions with at least 10k coverage. For this reason, our FHV vPAR-CL experiments reliably covered U34–U3034 on RNA 1, and U9–U1337 on RNA 2 (an example of coverage map can be found in Supplemental Figure S4). However, it is possible that we omitted potential capsid interaction sites out of our analyzed range. (ii) We previously noticed that certain point mutations may be selected by virus and could be associated with defective interfering RNA generation (37). In this study, we also noticed substantially increased mutation rates on certain genomic positions (such as U1259 on RNA1, as illustrated in Figure 2A–C). Thus, to eliminate virus intrinsic mutational events, we avoided direct use of U-C transition rate as a measurement. Instead, we decided to use fold change of U–C transition rate, between crosslinked virus and uncrosslinked virus control, as our vPAR-CL signals. (iii) To avoid the false positives due to low U–C transition rate in control groups, we introduced a background threshold whereby passing signals comprised the highest 5% of all U–C transition rates in control group (4SU+/UV–). In crosslinking group (4SU+/UV+), only the sites with transition rate above this threshold were taken into our further consideration, as they represent transition rates distinguishable from background fluctuation range (illustrated in Supplemental data 3). Together, we believe these three quality control measurements provided stringent analysis to our vPAR-CL data to reveal truly biologically relevant FHV RNA–capsid interaction sites.

### DMS-MaPseq and FHV secondary structure mappings

Several studies have proposed lowest free energy-based local RNA or whole genome secondary structure predictions for FHV (31,58,59,66). *In vivo* RNA chemical probing methods such as DMS and SHAPE allow for structure-specific chemical modifications to be screened by next-generation sequencing (43,67,68). Performing DMS-MaPseq in FHV virions, we are able to provide experimental validation of the RNA structures inside virus particles. With the same rationale of vPAR-CL, we also applied stringent quality control measurements to ensure reliable interpretation of mutational profiles generated by DMS-MaPseq: A/C error rates were only analyzed over positions with at least 10k coverage (A14-A3043 on RNA 1, and C11-A1378 on RNA 2); fold change of A/C mutation rate was regarded as DMS-MaPseq signals instead of actual mutation rate; similar background noise threshold was also applied to prevent potential false positives. Canonically, DMS-MaPseq data are imposed upon thermodynamic prediction by enforcing unpaired constraints on any position with a signal above a given threshold (43). In this study, we adjusted this approach by only allowing the most significant (top 5%) DMS-MaPseq signals to be unpaired constraints. However, one limitation in this study is that we constructed FHV secondary structural maps over RNA 1 and RNA 2 separately, omitting potential inter-RNA interactions.

Combining vPAR-CL and DMS-MaPseq, we demonstrated that these two high-throughput mutational profile technologies can work synergistically to answer basic virology questions. We observed that the FHV RNA–capsid sites heavily favored double stranded RNA structures in FHV RNA 1. This finding agreed with earlier predictions that the RNA duplexes scaffold the 2-fold axis of FHV capsid (19).

### Flock house virus vPAR-CL sites and biological implications

It has been observed previously that the RNAs of FHV, as well as other Nodaviruses, form a highly ordered dodecahedral cage inside virus particles (17,69). However, it was not clear whether the dodecahedral RNA cage had a fixed topology. From our vPAR-CL data (Figure 4A–D), we can clearly identify highly consistent RNA–capsid interactions over certain genomic positions among multiple replicates. This provides evidence that there is well-defined tropism between FHV RNA cage and capsid shell, at least at the sites identified here. Among the most consistent and distinctive vPAR-CL sites (Figure 4C, D), we noticed that they exhibited a highly clustered pattern. The clustering effect is more pronounced, when taking RNA secondary structures into consideration (Figure 6 and Supplemental data 1, 2). The multiple RNA–capsid interaction sites spanning the whole FHV genome suggests the possibility that FHV encapsidation may require multiple packaging signals to assemble the entire virus particle.

Several vPAR-CL sites also aligned with, or were adjacent to, known RNA motifs (Figure 8). On RNA 1 (Figure 8A), we could not align any candidate vPAR-CL signal to subgenomic RNA 3, which suggests the possibility that the exclusion of RNA 3 during packaging is due to lack of strong RNA–capsid interactions. Interestingly, two most significant vPAR-CL sites on RNA 1, U159 and U1233, aligned with previously discovered replication regulatory elements: a 5′ cis element (nt. 68–205) that is essential for RNA 1 replication and mitochondria-targeting (58), and short distal subgenomic control cis-element (nt. 1229–1239) which mediates subgenomic RNA 3 replication (59). Furthermore, U2515 and U2576 were located in the subgenomic promoter region (59,70) which are also adjacent to a RNA 1 internal cis-acting replication element (intRE, nt. 2322–2501) (59). Similarly, on RNA 2 (Figure 8B), we noticed vPAR-CL site U534 is adjacent to a RNA 2 cis-acting regulatory site (71), and U1155 which is within a site required for specific packaging of both RNAs (25). A previously predicted stem loop site (nt. 168–249) on RNA 2 serves as a RNA 2 packaging signal (31). This is also the only established FHV RNA packaging signal to date. Our DMS-MaPseq map did predict a near identical stem loop structure as previously proposed and we noticed three significant (>1σ) vPAR-CL sites were clustered in this critical region (Supplemental Figure S11). Since these RNA–capsid interaction sites coincided with RNA cellular replication/mitochondrial targeting sites, we suggest they might be multi-functional in the virus life cycle, and there can be a strong synergy between protein A-mitochondria localization (16,72,73), RNA replication, and virus assembly.

It was speculated previously that FHV packaging and replication are coordinated events. When FHV and brome mosaic virus (BMV) were co-expressed in plant cells, assembled virions only packed their own respective viral RNAs (27). Intracellular protein-protein interactions between FHV replicase (protein A) and capsid were detected (28). It has also been shown that FHV can ensure genome assembly specificity only when capsids were translated from replicating viral RNAs (30). It was hence suggested that FHV encapsidation may be coupled with the RNA replication. Our FHV vPAR-CL experiments directly implicated only one aspect of FHV biology: the RNA sites interacting with capsid proteins within virus particles. However, upon further analysis and mutational assays, a number of vPAR-CL sites clearly had important roles in FHV replication and regulation: U159 and U227 mutants showed severe deficiency of RNA 1 replication and virion production, while U1233 entirely abolished viral replication. This provides further evidence that FHV replication and packaging are not sequentially separated events, but rather are synchronized, inter-dependent processes. Furthermore, these RNA–capsid interactions are not only important in post-replicational/translational RNA packaging but may also be essential for multiple aspects of virus early stage activities in host cells. It is important to note that the in virion capsid–RNA interactions may be different from the intracellular interactions between capsids and viral RNAs in host cells. Whether these in virion interactions are conserved during cellular packaging process is still unclear. Future intracellular studies will confirm whether these vPAR-CL sites are related to the specific selectivity of FHV RNAs by capsid proteins.

Overall, our studies and vPAR-CL methodology provide insight into the nature of the encapsidated RNA genome of ssRNA viruses. Some ssRNA viruses, such as MS2 phage, have successfully yielded asymmetrical cryo-EM reconstructions that reveal the internal topology of the packaged genome (1). For other viruses that have to date eluded asymmetrical reconstruction, such as FHV when unperturbed by partial disassembly (74), our approach demonstrates that the encapsidated genome does indeed contain regions of coherent and consistent structure rather than being packaged in an asynchronous and disordered fashion. Future modeling and structural analyses will confirm whether there exists a singular topology or an ensemble comprising partially disordered regions together with conserved structured regions. vPAR-CL methodology may easily be deployed in other ssRNA viral systems, as well as under experimental conditions which perturb internal RNA structures (such as virus particle disassembly).

## DATA AVAILABILITY

The raw sequencing data for both vPAR-CL and DMS-MaPseq experiments are available in the NCBI sequence read archive (SRA) with accession number: PRJNA554838.

## Supplementary Material

gkz1124_Supplemental_Files
