# Supplementary material for: Mapping RNA–capsid interactions and RNA secondary structure within virus particles using next-generation sequencing
Source: Nucleic Acids Res. 2019 Dec 4;48(2):e12. doi: 10.1093/nar/gkz1124 (PMC6954446; doi:10.1093/nar/gkz1124)
Supplement: gkz1124_Supplemental_Files [file gkz1124_supplemental_files.zip › Supplemental Figures_revision.pdf]

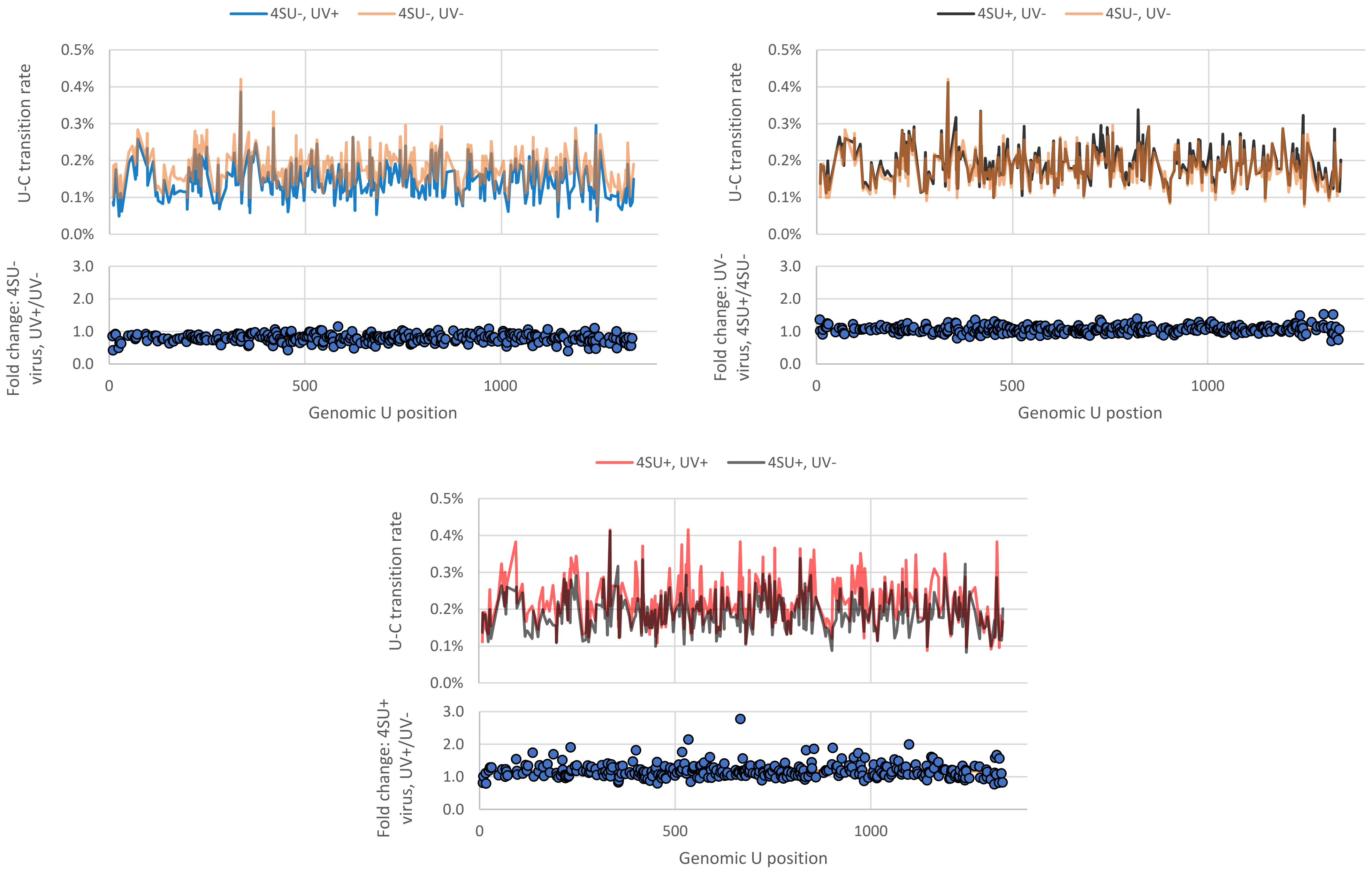

Supplemental Figure S1a

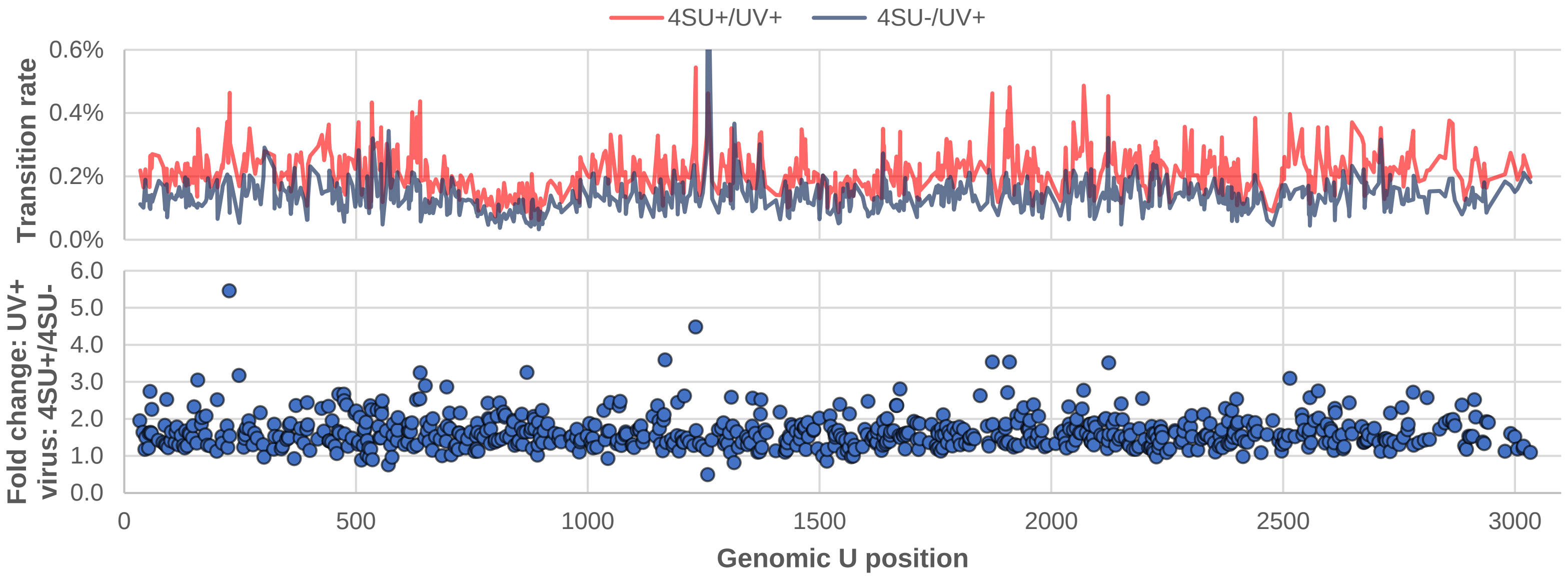

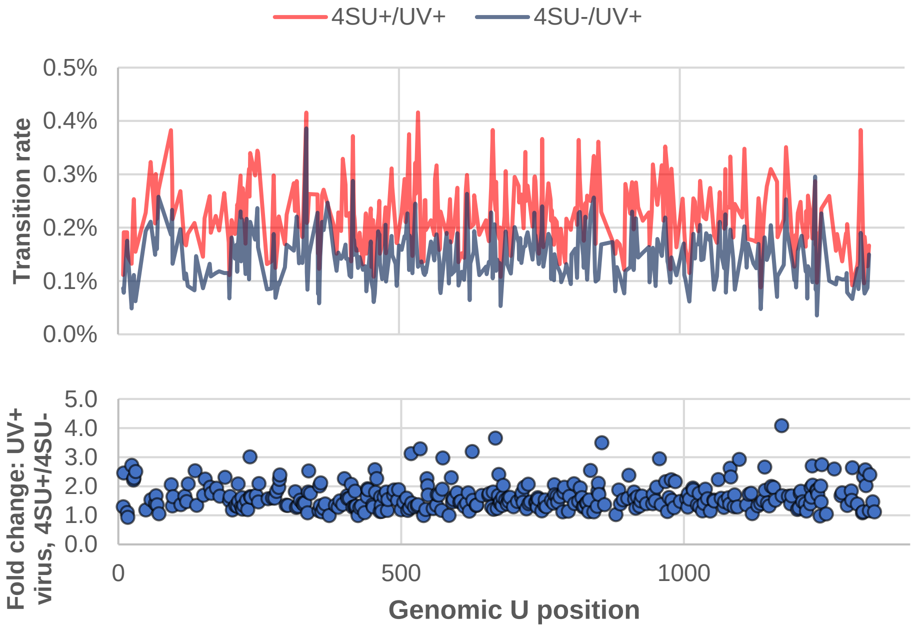

Supplemental Figure S1b

Supplemental Figure S1. “U-C” transition rate elevating is specific for crosslinking events.

(**a**)In RNA 2, we observed the same phenomenon as that in RNA 1: UV irradiation or 4SU substitution alone was not sufficient to induce substantial U-C transition rate change. Only when UV and 4SU substitution both presented, did we observe increased vPAR-CL signals over certain sites. **(b)** When comparing 4SU+/UV+ and 4SU-/UV+, we observed substantial fold change. This is likely related to the reduced U-C transition rate in 4SU-/UV+ virus.

Experimental condition	Priming 4SU cct.	Boosting 4SU cct.	Final cct.	Incubation time
**4SU16h**	100uM (0hpi)	-	100uM	16 hpi
**4SU1.5X**	150uM (0hpi)	-	150uM	16 hpi
**4SU40h**	100uM (0hpi)	100uM (16hpi)	200uM	40 hpi

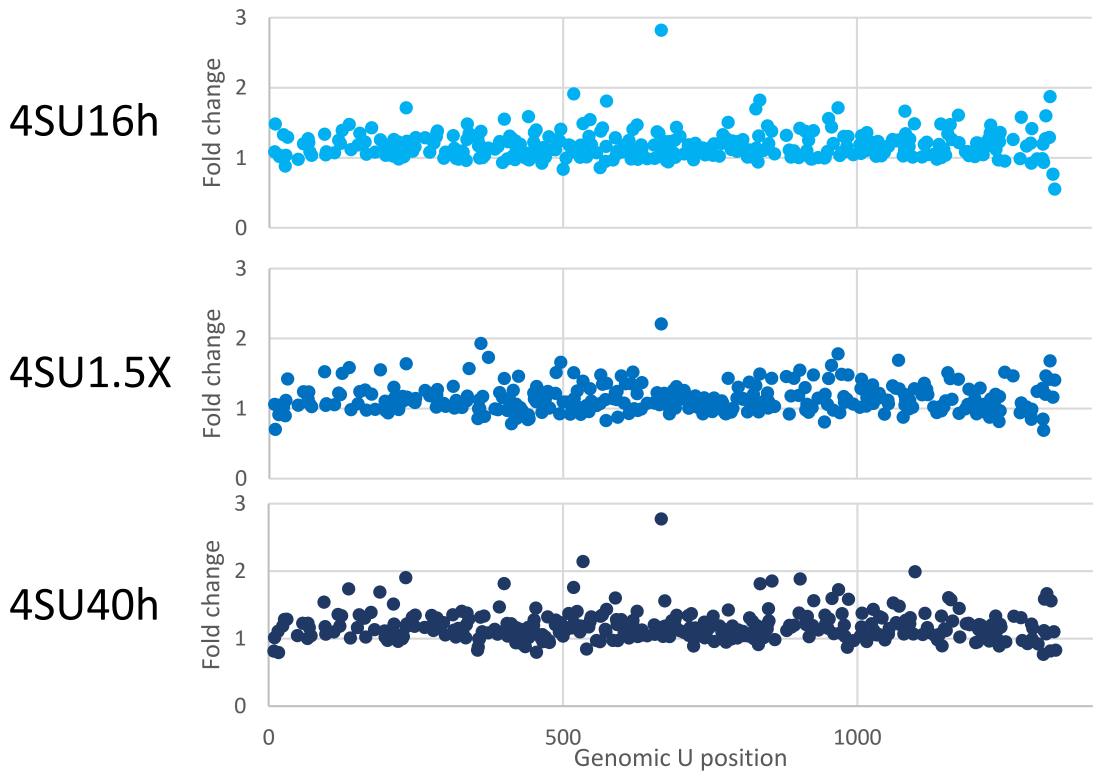

Supplemental Figure S2a

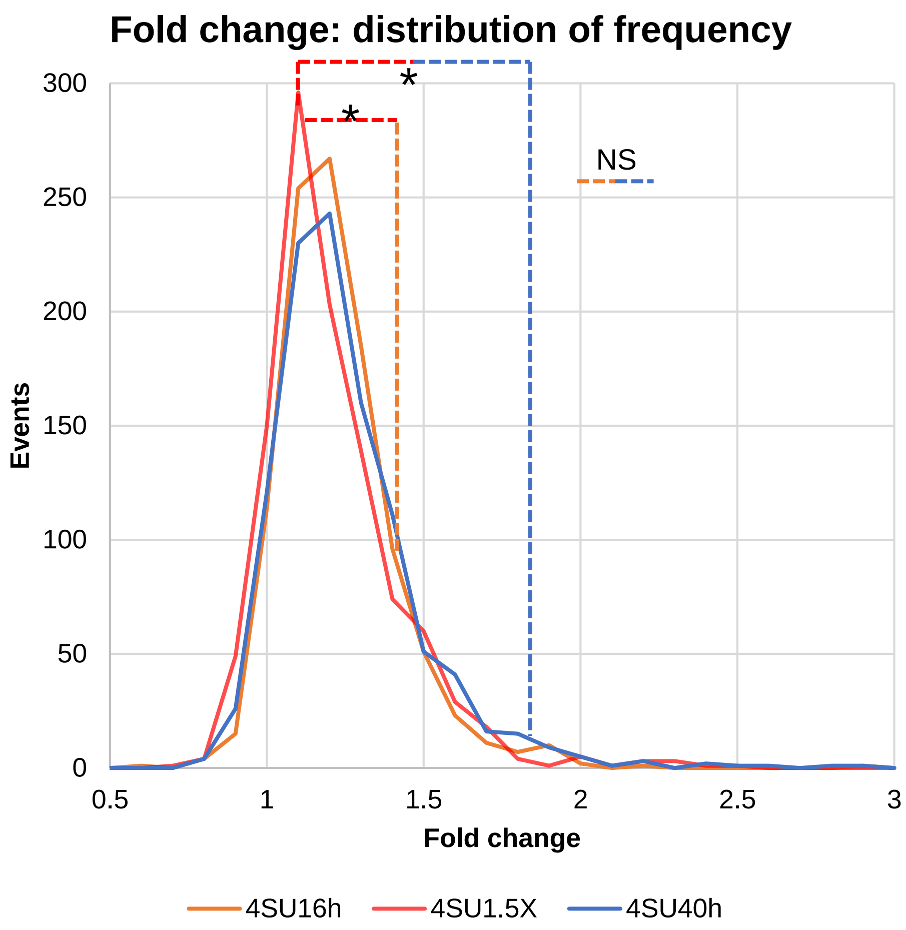

Supplemental Figure S2b

Supplemental Figure S2. vPAR-CL signal intensities were correlated with 4SU dose and incubation time.

(a) Similar to RNA 1, in RNA 2, we also noticed the intensity of vPAR-CL signals correlated to the dose of 4SU and time of incubation, with 4SU40h gave the best result. **(b)** Statistical assays conducted to compare distribution of vPAR-CL signals under different conditions (Two-Sample Kolmogorov-Smirnov Test, *: $p<0.05$; NS=not significant).

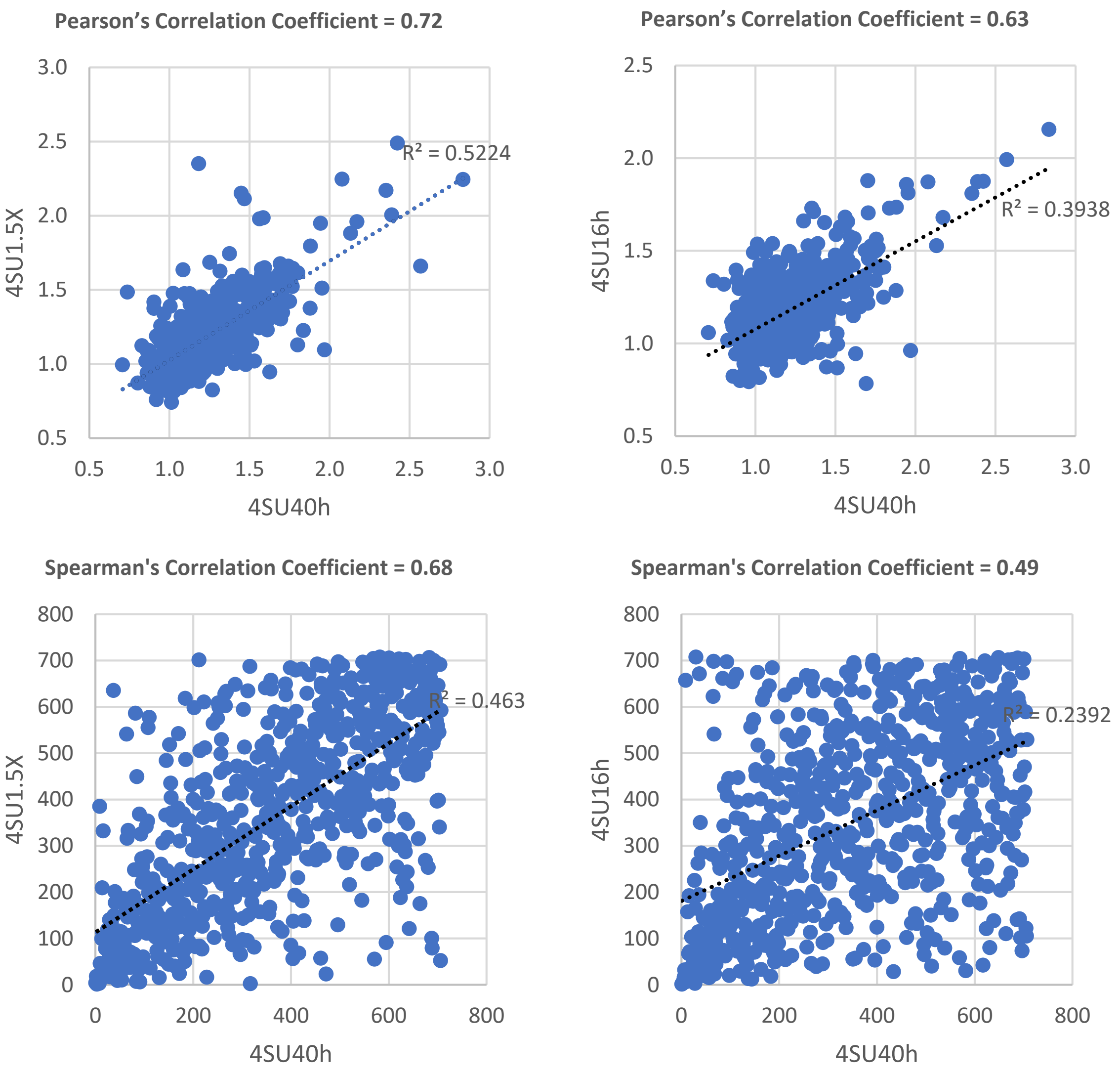

p=3E-115 *p*=1E-78

Supplemental Figure S3

Supplemental Figure S3. Good correlation coefficient of vPAR-CL signals under different experimental conditions.

Despite varied signal intensities, we observed good (≥0.6) Pearson's correlation coefficient between 4SU40h, 4SU1.5X, and 4SU16h experiments, indicating reproducibility of vPAR-CL experiments. Regression Statistics were conducted with p-value calculated for each correlation. Spearman's correlation coefficient tests are also included.

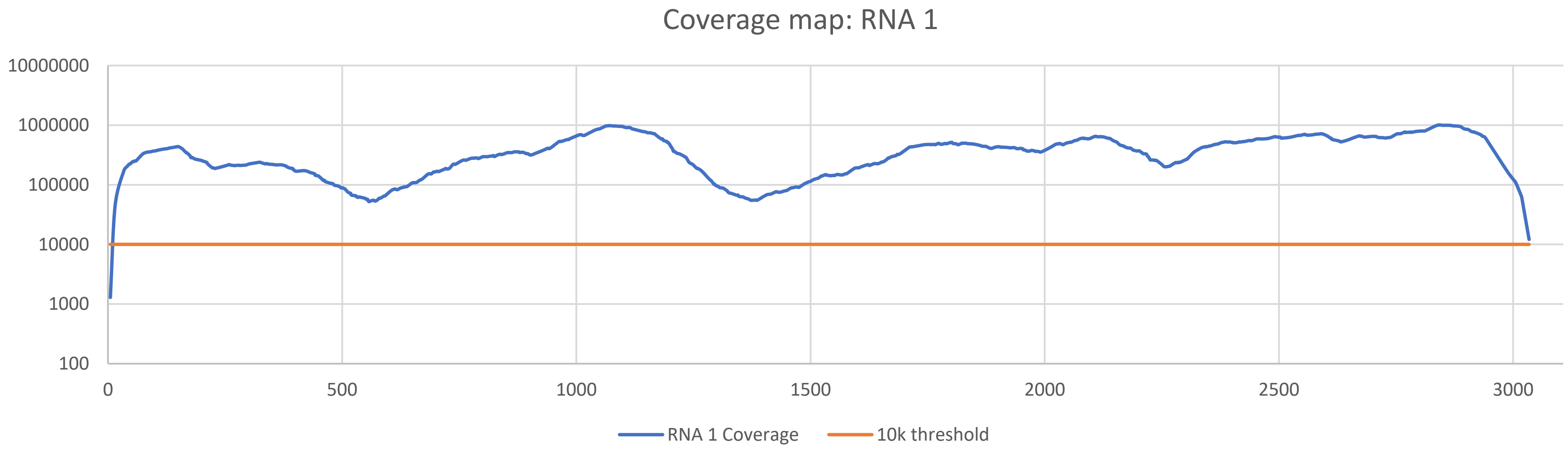

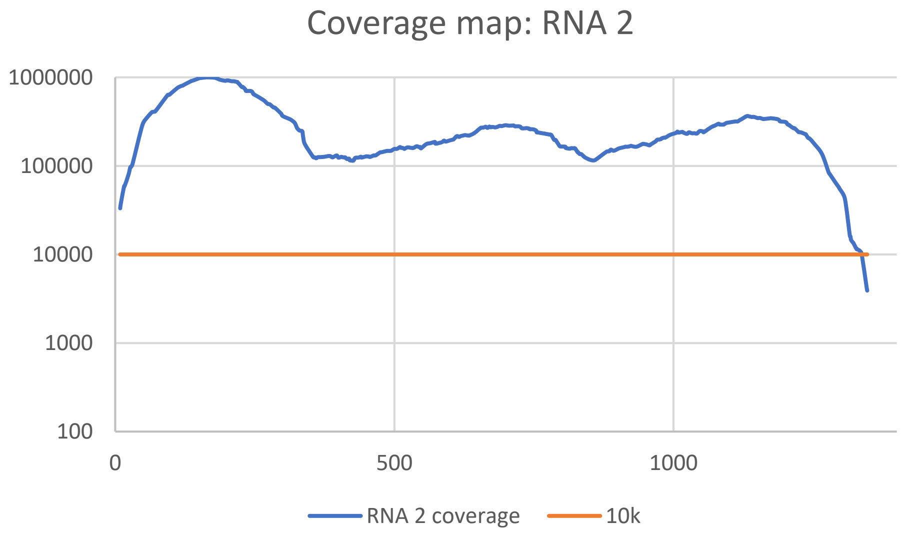

Supplemental Figure S4

Supplemental Figure S4. Sequencing coverage.

After vPAR-CL experiment, the 4SU+/UV+ viral RNA was sequenced with ClickSeq. As illustrated for RNA 1, ClickSeq achieved relatively even coverage except for 5’ and 3’ termini of RNA genome. In order to ensure sufficient depth of coverage and stable transition rate, we selected 10k coverage as a threshold. Only the individual bases with coverage above this threshold were used of vPAR-CL signal calculation (RNA 1: U34-U3034; RNA 2: U9-U1337).

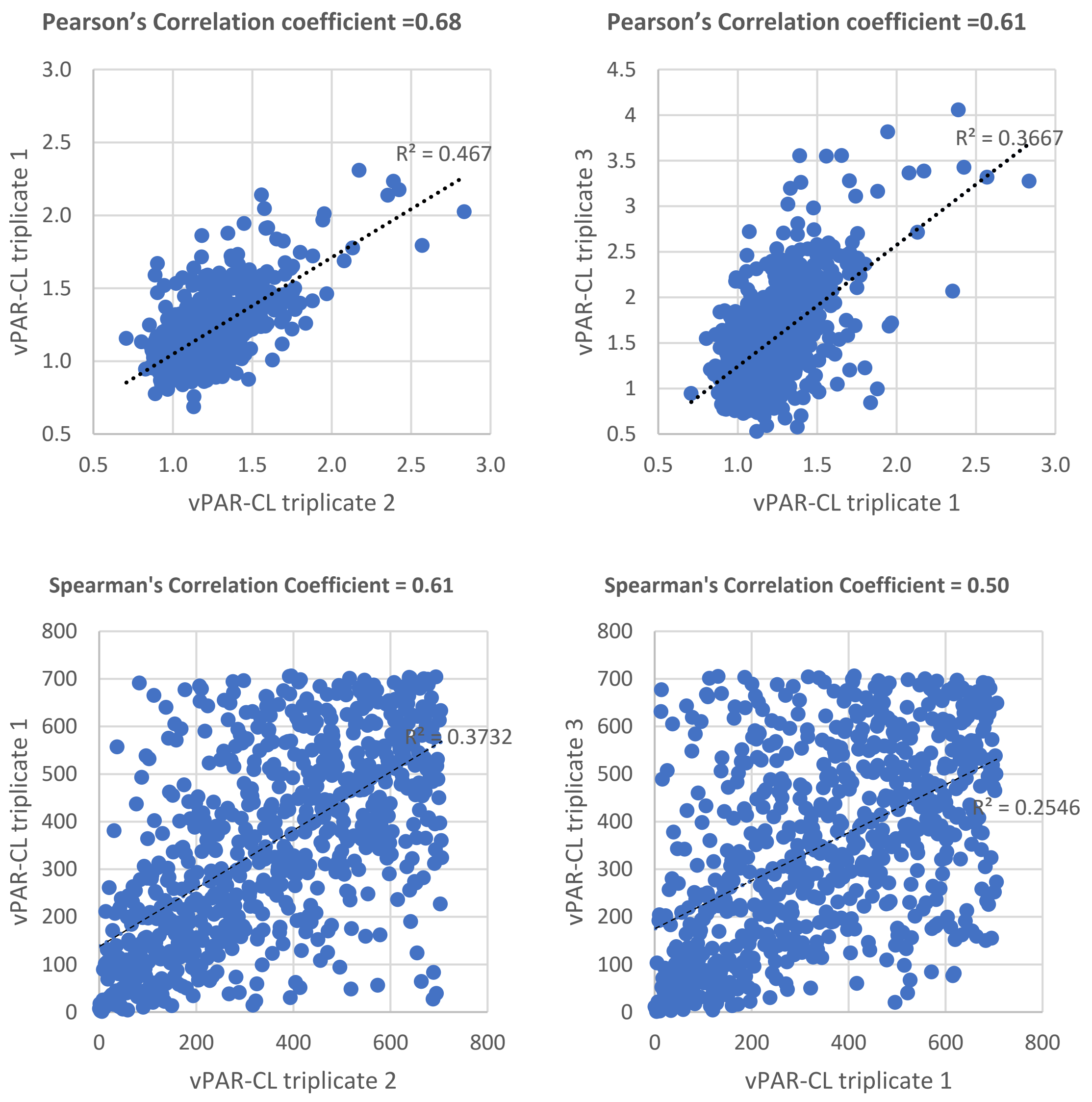

Supplemental Figure S5

Supplemental Figure S5. Good correlation coefficient of vPAR-CL signals under FHV vPAR-CL triplicate.

Despite varied signal intensities, we observed good (≥0.6) Pearson’s correlation coefficient between FHV vPAR-CL triplicate, under 4SU40h condition. This indicates reproducibility of vPAR-CL experiments. Spearman’s correlation coefficient test are also included.

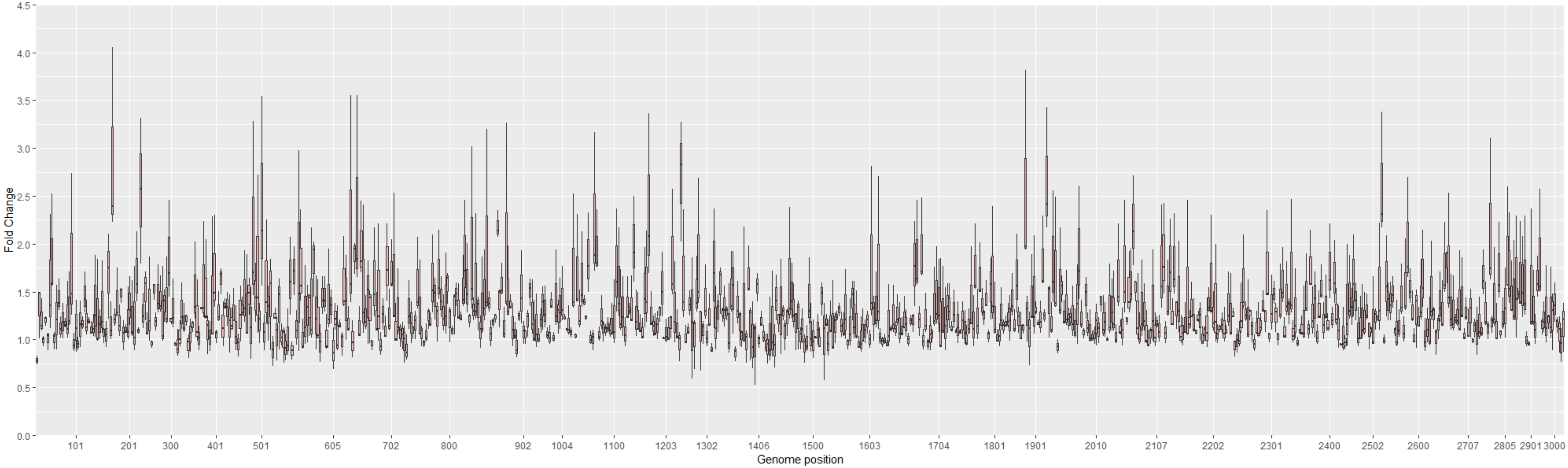

a

Supplemental Figure S6

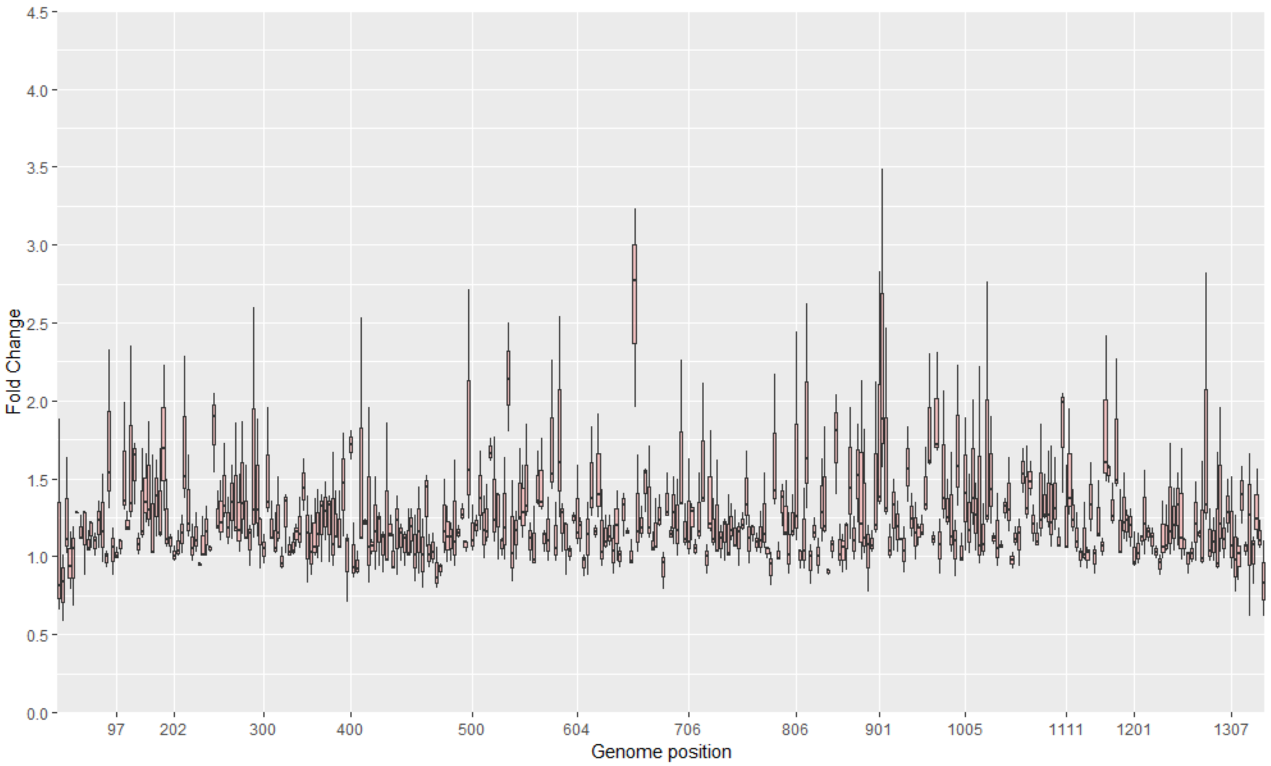

b

Supplemental Figure S6

Supplemental Figure S6. Triplicate FHV vPAR-CL signals.

Triplicate FHV vPAR-CL signals were box-plotted for RNA 1 **(a)** and RNA 2 **(b)**, respectively, without background threshold filter. X-axis is not continuous.

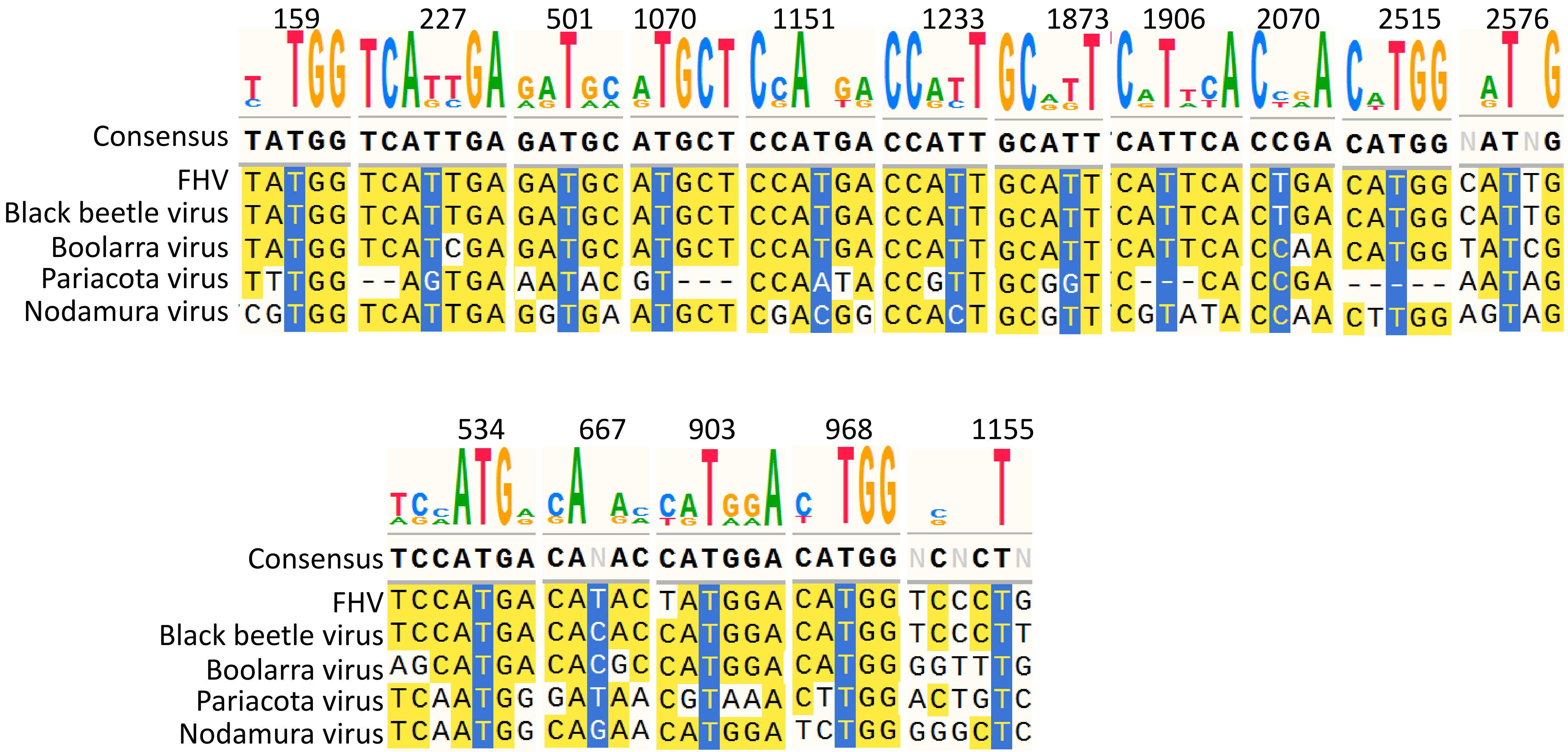

Supplemental Figure S7

Supplemental Figure S7. Sequence conservation of vPAR-CL sites.

MUSCLE (Multiple Sequence Comparison by Log-Expectation) was used to align FHV RNA 1 and 2 sequences with other validated Alphanodavirus sequences. Most of the sampled vPAR-CL sites showed good sequence conservation.

U position	Flanking sequence (101nt.)
159	GUAUCUGGGUGCGGUGCCGUAGUGUACUGCAUAUCCAAGUUCUGGGGCUAUGGGGCAAUUGCGCCCUAUCCUCAGAGUGGAGGGAACCGAGUUACACGCGC
1233	GUGUCACCAAUUAGUCCAACUGUGUAUAAACCUACAAUGCCACGCGUCCAUUGGCCAGUAACCAGUGACGCAGAUGUACCAGAAGUGAGCGCGCGCCAAUA
1910	CAUGGUUCAAGCAUUCCGCCCAGAAUACAGAGAUGAGAUCAUUUCAUUCAUGGACACGAUAAUCAAUUGUCCAGCUAAAGCUAAACGCUUUGGUUUCCGAU
2515	GUAGAAGCCGGAAUAAAGAGAAGCCCUACUGGUUGACUUGUGACGGAUCAUGGCCACAGCAUCCGCAAGACGCCCAUUUGAUGAAGCAGGUUUUAAUCAAA
1873	UUUCCAGCUGGAUGCAAAGAAACAUCGCCCAAAAGGCCAUGGUUCAAGCAUUCCGCCCAGAAUACAGAGAUGAGAUCAUUUCAUUCAUGGACACGAUAAUC
227	UCCUCAGAGUGGAGGGAACCGAGUUACACGCGCAUUGCAACGGGCUGUCAUUGACAAAACGAAGACCCCGAUAGAGACACGUUUCUAUCCGCUUGACAGCC
501	GCCGUCGGUGAUUUGGCACAAGAUUUCCGUAAUGACACACCUGCGGAUGAUGCCUUCAUCGUCGGUGUUGAUGUUGAUUAUUAUGUCACCGAGCCUGAUGU
1167	GGUAUGGGACACAAGGACCCGCAAUACACAUCCAUGAUUGUCCAGUAUUAUACUGGCAAGAAGGUAGUGUCACCAAUUAGUCCAACUGUGUAUAAACCUAC
639	UUUAACCCGAAGAAAGUGAGCGGUUUUGAUGCUGACUCACCAUUCACCAUUAAGAACAACUUGGUUGAAUAUAAGGUUAGCGGUGGAGCAGCAUGGGUCCA
1068	GGUCGAGAAGGGGAGCAUGCUCAGAUUACUAUCGAGAAAGAAAAGUUGGAUAUGCUCUCGGGAUUAUCCGCCACCCAAUCUGUCAACGCUAGGCUUAUCGG
2070	UACAAUGGAUGUGUCGAAUUUACAGCUCUGACCUUUGAGCAUCCUGAUGCUGAACCUGAAGAUUUGUUCCGUUUAAUCGGACCGAAGUGCGGUGAUGAUGG
869	UCAUUGUAGACCGUGGACUGAUUGUCCAGAUCGUGCACUUGUCUACACUAUACCGCAAUAUGUCAUUUGGCGAUUUAAUUGGAUUGAUACCGAACUACACG
631	UACACACCUUUAACCCGAAGAAAGUGAGCGGUUUUGAUGCUGACUCACCAUUCACCAUUAAGAACAACUUGGUUGAAUAUAAGGUUAGCGGUGGAGCAGCA
2757	UCCGUGGACGAAUCGUUACCAAUGUUAAACGAUGCCAAGCAAACUCGCGCUAAUCCAGGAACUUCCCGACCGCAUUCAAACGGCGGUGGAAGCAGCCAUGG
480	CUGCGAAACCAUUUCCACUUCGCCGUCGGUGAUUUGGCACAAGAUUUCCGUAAUGACACACCUGCGGAUGAUGCCUUCAUCGUCGGUGUUGAUGUUGAUUA
2576	UCCGCAAGACGCCCAUUUGAUGAAGCAGGUUUUAAUCAAACGUACAGCCAUUGACGAAGAUCAGGUCGAUGCACUCAUUGGGCGUUUUGCCGCAAUGAAGG
884	GACUGAUUGUCCAGAUCGUGCACUUGUCUACACUAUACCGCAAUAUGUCAUUUGGCGAUUUAAUUGGAUUGAUACCGAACUACACGUGCGAAAACUGAAAC
1151	CAACGCUAGGCUUAUCGGUAUGGGACACAAGGACCCGCAAUACACAUCCAUGAUUGUCCAGUAUUAUACUGGCAAGAAGGUAGUGUCACCAAUUAGUCCAA
650	GAAAGUGAGCGGUUUUGAUGCUGACUCACCAUUCACCAUUAAGAACAACUUGGUUGAAUAUAAGGUUAGCGGUGGAGCAGCAUGGGUCCAUCCAGUUUGGG
857	UCAUAAAAUCCAUCAUUGUAGACCGUGGACUGAUUGUCCAGAUCGUGCACUUGUCUACACUAUACCGCAAUAUGUCAUUUGGCGAUUUAAUUGGAUUGAUA
1070	UCGAGAAGGGGAGCAUGCUCAGAUUACUAUCGAGAAAGAAAAGUUGGAUAUGCUCUCGGGAUUAUCCGCCACCCAAUCUGUCAACGCUAGGCUUAUCGGUA
832	UGAUUGGUUUGGAGAAGGUUGGCUAUCAUAAAAUCCAUCAUUGUAGACCGUGGACUGAUUGUCCAGAUCGUGCACUUGUCUACACUAUACCGCAAUAUGUC
1674	UUUCCAGACAUACUUUUCAUCUUGAAAGUUUCCAGAUACACCUUAGCGUAUUCGGAUAUAGUUCUACAUGCCGAACACAAUGAACAUUGGUAUUACCCCGG
1962	GACACGAUAAUCAAUUGUCCAGCUAAAGCUAAACGCUUUGGUUUCCGAUAUGAGCCUGGUGUAGGCGUUAAAAGUGGAAGUCCAACAACCACGCCACAUAA
2811	CCAGGAACUUCCCGACCGCAUUCAAACGGCGGUGGAAGCAGCCAUGGGAAUGAGCUACCAAGACGCACCGAACAACGUGCGCAGGGACCUCGACAACCUGC
56	CGAAACAAAUAAAACAGAAAAGCGAACCUAAACAAUGACUCUAAAAGUUAUUCUUGGAGAACACCAGAUCACCCGAACUGAAUUGUUAGUCGGGAUUGCAA
2118	GCUGAACCUGAAGAUUUGUUCCGUUUAAUCGGACCGAAGUGCGGUGAUGAUGGUCUUUCCCGGGCUAUCAUUCAAAAAUCAAUUAAUCGCGCUGCCAAGUG
1667	AUCCGGUUUUCCAGACAUACUUUUCAUCUUGAAAGUUUCCAGAUACACCUUAGCGUAUUCGGAUAUAGUUCUACAUGCCGAACACAAUGAACAUUGGUAUU

U position	Flanking sequence (101nt.)
667	ACUGUGCAAUUCCCGGUUGCAACAGAUCCAGCCACCAGUUCGCUAGUUCAUACUCUUGUUGGUUUAGAUGGUGUUCUAGCGGUGGGGCCUGACAACUUCUC
1318	ACCGGGCAAGUAUCCGAACAAUCGGACAUUUGGCCACAAUAAGCCCAAUUUGGUUGAAGAUUAAAGUAGUGAGCCCCCUUAGCGCGAAACCGGAAUUUAUA
903	CAUGGACUCAGGAGCAGAAGCCACCAGUGGAGUAGUCGGAUGGGGCAAUAUGGACACGAUUGUCAUCCGUGUCUCGGCCCCUGAGGGCGCAGUUAACUCUG
534	AACUUCAACAUCUAGGUCCGAUCAGGUGUCCUCAUUCAGGUACGCUUCCAUGAACGUGGGUAUUUACCCAACGUCGAACUUGAUGCAGUUUGCCGGAAGCA
968	UCCGUGUCUCGGCCCCUGAGGGCGCAGUUAACUCUGCCAUACUCAAGGCAUGGUCCUGCAUUGAGUAUCGACCAAAUCCAAACGCCAUGUUAUACCAAUUC
817	GACUUUGAAUUCAAUGACAUAUUGGAGGGUAUCCAGACAUUGCCACCUGCUAAUGUGUCCCUUGGUUCUACGGGUCAACCUUUUACCAUGGACUCAGGAGC
901	ACCAUGGACUCAGGAGCAGAAGCCACCAGUGGAGUAGUCGGAUGGGGCAAUAUGGACACGAUUGUCAUCCGUGUCUCGGCCCCUGAGGGCGCAGUUAACUC
496	CCCGUUAAUUAUCCGGGUUUUACAUCGAUGUUCGGAACAACUUCAACAUCUAGGUCCGAUCAGGUGUCCUCAUUCAGGUACGCUUCCAUGAACGUGGGUAU
957	CACGAUUGUCAUCCGUGUCUCGGCCCCUGAGGGCGCAGUUAACUCUGCCAUACUCAAGGCAUGGUCCUGCAUUGAGUAUCGACCAAAUCCAAACGCCAUGU
1155	CCAAAAUGCAUCAAUGUGGGAGAGAGUGAAAUCCAUCAUUAAAUCCUCCCUGGCUGCUGCAAGCAACAUUCCCGGCCCGAUCGGUGUCGCCGCAAGUGGUA
233	CGGCGCUAACCAGAUUAAGUCAACCUGGUUUGGCGUUUCUCAAAUGUGCAUUUGCACCACCUGACUUCAACACCGACCCCGGUAAGGGAAUACCUGAUAGA
1098	CGCGCUUCAGGAAUACCGUACGGUUGCCAGAUCUUUGCCGGUUGCAGUGAUAGCGGCCCAAAAUGCAUCAAUGUGGGAGAGAGUGAAAUCCAUCAUUAAAU
589	GUGGGUAUUUACCCAACGUCGAACUUGAUGCAGUUUGCCGGAAGCAUAACUGUUUGGAAAUGCCCUGUAAAGCUGAGUACUGUGCAAUUCCCGGUUGCAAC
1266	GUCAGCCCUUUUUGAAGGAUUUGGCUUUUAGAAGCAUCCGGACGCCAACCUAACCGGGCAAGUAUCCGAACAAUCGGACAUUUGGCCACAAUAAGCCCAAU
835	AUAUUGGAGGGUAUCCAGACAUUGCCACCUGCUAAUGUGUCCCUUGGUUCUACGGGUCAACCUUUUACCAUGGACUCAGGAGCAGAAGCCACCAGUGGAGU

Supplemental Figure S8

Supplemental Figure S8. DREME analysis resulted in no common sequence among vPAR-CL sites. The flanking sequences of 28 RNA 1 vPAR-CL sites and 15 RNA 2 sites are listed.

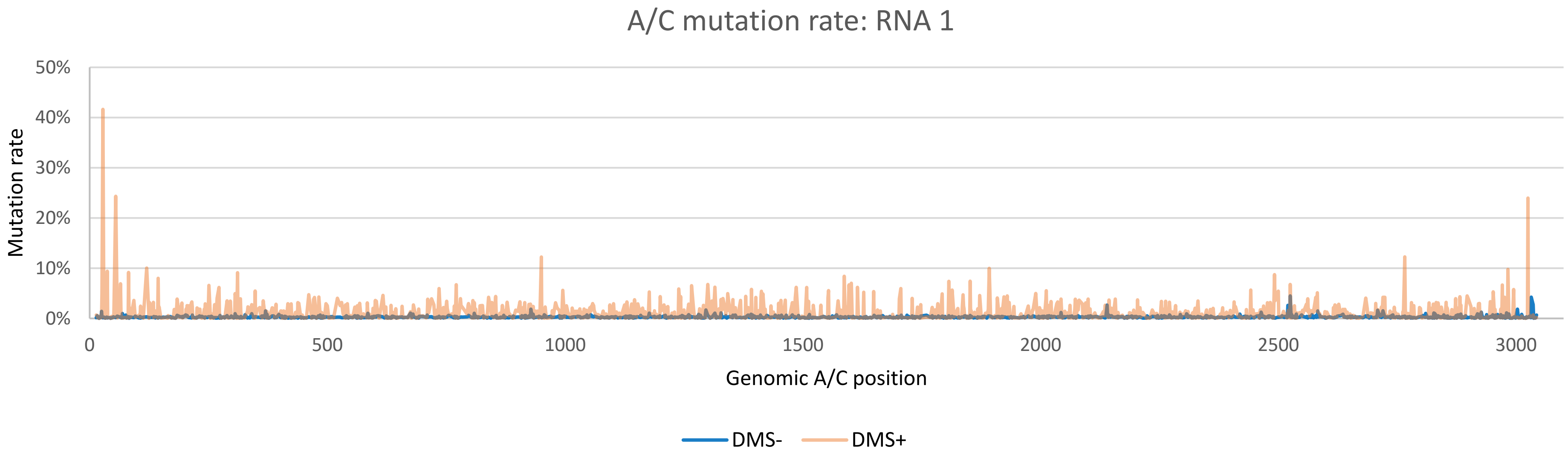

Supplemental Figure S9a

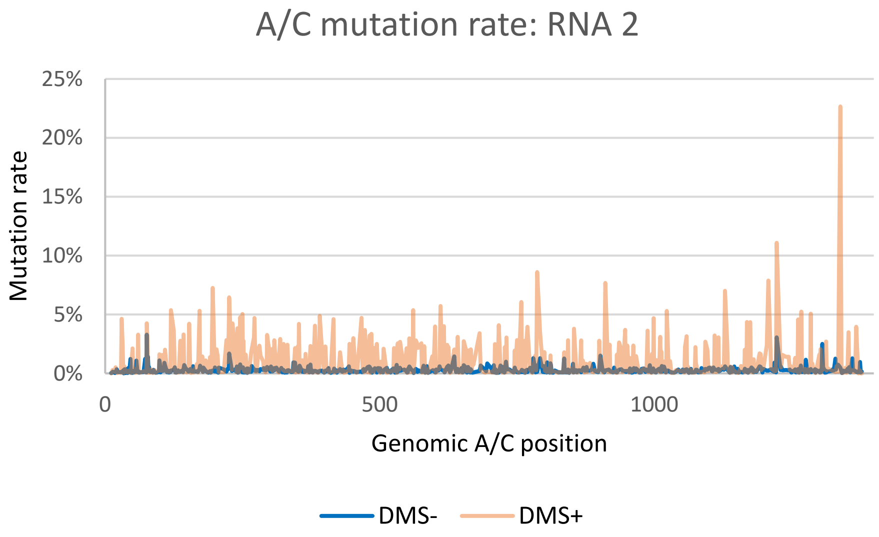

Supplemental Figure S9b

DMS-MaPseq signal : RNA 1

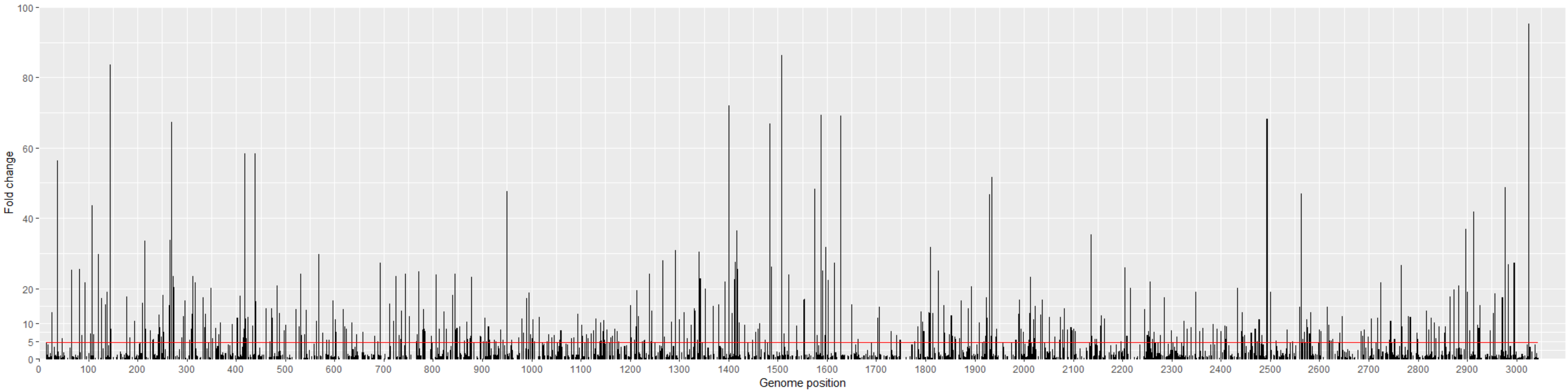

Supplemental Figure S9c

DMS-MaPseq signal: RNA 2

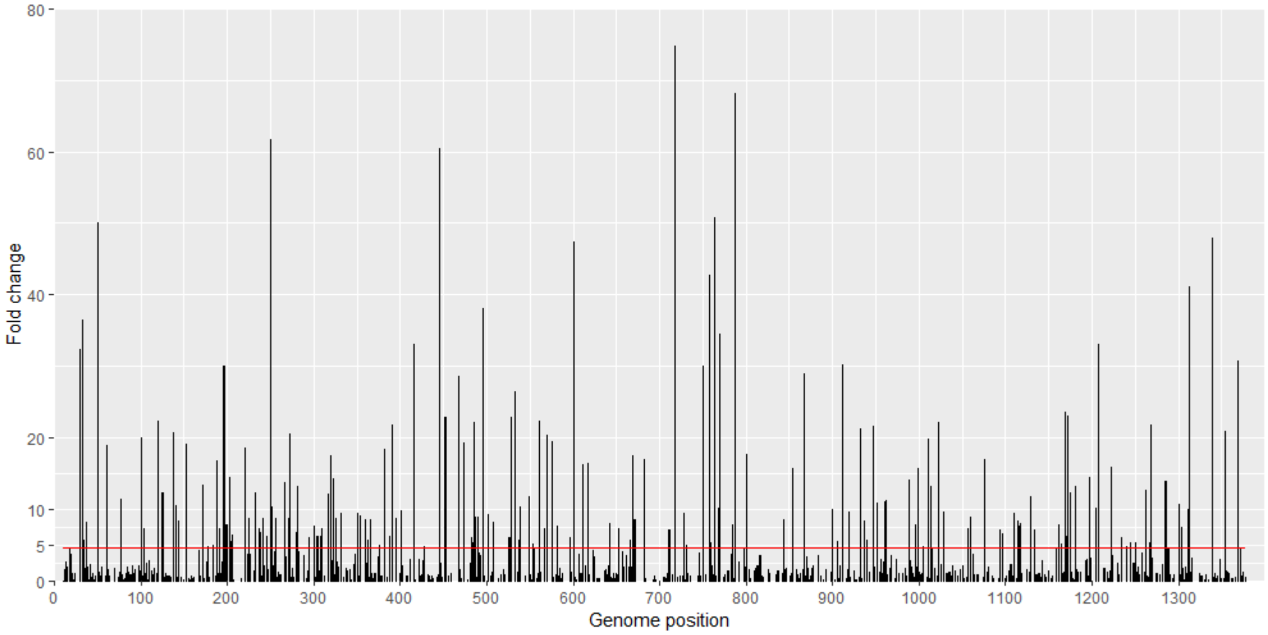

Supplemental Figure S9d

Supplemental Figure S9. FHV in virion DMS-MaPseq.

(a,b) Error rates for adenine and cytosine positions are compared between DMS-treated virus (DMS+) and untreated virus (DMS-). **(c,d)** DMS-MaPseq signals represent the fold change of A/C mutation rates between DMS-treated virus and untreated virus.

RNA 1

>ENERGY = -1019.7 RNA1_ no constrain

```
..((((..............)))).(((.........((((((.....))))))(((((.....((((((((((((.((((....)))))))))..)).......))))))((((((((......)))))))).)))))).((((((..((((((......))))
)))))))))((((.(((...((((((......((((((...(((((((((.................((((((((....))))))).))..)))))))))((((((((.(((((.((((...(((((....(((((..((((((((.((((....((((((..((.
(((((......))))).)).)))))))))..(((.((((.......((((((((((((((.((((..(((.(((.(((..((.(((((((((.((....(((((.((((((...((((((.....((((((..........)))))))).)))).))))))).))))).((((
((........)))))....((...((((((((...)))))))))).((((....((((...((((.((((((........((((((((.....)))).))))).((((((((............))).)))))....)))))).)))).))))...))))....((((((
.((...(((.((...((((((((.....))))))))...))))).)))))))))).))))))))))..))....))).)))...))))))).))))....)))))))).)))....)))).))).((((((.(((.((.((((.....)))))).)))..)))))))).....
.((((.(.(((..((((((((((....))))))...))))).)))..))))).....)))))))))..))...((((((...........)))))).((.....(((.((((((((..((.........(((.(((.(((...))).))))))......))..)))))))).))).
(((.((......))..))).(((((..((.....))..))))).)).)))..))))).))))))))))).))))))..(((((.((((.......((((..((.....(((.((((((...))))))))...))..)))).........)))...)))))..........))
))))....((((((.(((((((((..(((..((((((.(((((((..(((((((.(((((..(((((...((.(((((.((....((((((..((((((((((.(((.(((.((......)).)))((.((((((((((.((.((((((((.((((((((...((.........
.)).)))))))))))))).....((((((.((((((.((.(((((((((((.(((.((...........)).)))))))))))))).)).)))))).))...))))))....((((((((.................)))))))).((.((....)).))......)))))))))))
))).))(((((((.....)))))..((((((........(((((((...)))))))))))))).....((((((.....)))))).(((.(((((........))))).)))....((((.........))))....))).)))))))))))....((((((..(((.(((((
(((((((.(((.(((.(.(((...((((((..(((((((......)))))))..))))))..))))))).))).)))).)))))..))))).))).))).(((...((..(((.(((.....))).)))..))..))).)))))))...))))))).((((((...))))))..((((
(..........)))))(((((((((.....))))))))).)))))))...))...)))))))))).....((((..((...))..)))).(((((..((((..........((((((((((...((.(((((.(((..((((((.........(((((..(((....((((.((
((......)))).))))...))).((((((((((((.....)))))))))))...............(((((....))))).))))))))))).))).))))))))))))).)))).((((((((.........)))))))))))...)))))...)))))))......((..
.....)).......))).))))))))))...))).....))))........(((((.((((.((((.(((.((((((((........))))))))..((((.((((((((((((.((((..((.((((........)))).)).))))...))))))..((.((((((((((((((
((((.....))))).))).))..))..))))).)).))......)))))))))))).))))....))))...)))))).))))).(((((((((((((((((((((.((((((((((....)))).)))))).......((.((((((.........)))))))......)))))))
)))))..........))))).)))((((.((((...(((((.(((((..........)))))))))))..))))........(((((...))))).)))))))))).((((((.........))))))((...)).)))))).(((..((((((..(((((..(((((.....
....))))))))))...)).))))....)))((((((.(((..((((......(((.((((((...........))))).))).(((((.....)))))........))))..)))...))))).((((((.(((((......)))))))))))...........((........))
)..((((((....................))))))....((((..((...))..))))((((((........)))))....))))))).)))......(((.((((.(((....))))))).))).((..(((.((((....)))))))).
```

>ENERGY = -896.6 RNA1_DMS-MaPseq corrected

```
..((((..............)))).((((.........((((((.....))))))).((((.......))))....(((((.(.((....)).)))))).))))..........((((.(((((....((((.(((..........((((((...((((((......))))
))).))))))).((......))...................((((..(((.((((......))).))))))).)))..))))...................((((.......((((((.......)))))))......))))).((((.........)).))..((((((..((.
(((((......))))).)).))))))).(((((((.((((((...((((.(((........)))))))).......((((((.(((((...........))))).)..(((((....((((((.....((((((..........)))))))).)))).))..)))))).(((((.....
..............)))))......((((((((...))))))))..)))))).((((...((((.((((((........((((((((.....)))).))))).((((((((...........))).)))))....)))))).)))).....)))))))))))).......
..(((((((((((.((((((((.....))))))))...(((((....((.(((.((..(((.....))..)).)))))(((..((((((((((...(((...(((((.....(((..(((((((.((((((.(((.((..............)).)))..)))))).....
.((((.(.(((..((((((((((....))))))...))))).)))..))))).......((((((.((((...((((.....(((((....))))).((.((((((((.((.(((......))).......((.(((.(((...))).)))))......((((((((.(((....
(((.((......))..)))))).))))))))))(((....)))...)))))))))))..((((((((((((((...(((........)))..........((((((((.((((((...)))))))))...(((.(((((((..............(((.(((((...((...
......(((((.(((((.......)))))))))))).))))))))..)))).)))..))).((((((((((...........)))).)))))..(((((((((((.((((...((((((....((((((((.....((...(((..((.....((((((((.......)))))))..
))..)))...)).((((.......).)))..(.(((.((.((((((((((.(((.((..............)).)))))))))))).)).))).).((((((.(((.((((((((((.((..................)))))))))))......(((((((.....)))..))))).
))).))).)))))).........(((((......)))))(((((((...)))))))..))).)))))...)))))).)))))..((.(((.(((((........))))).))).)).((((.........))))........)))))))))))....((((((((.(((.(((((
((((((.(((...((.(((...((((((..((((((((......))))))..))))))..)))))))..))).)))..))))).))).)))......((..(((.(((.....))).)))..))....)))))))...........(((((...))))).)))))).
(((((.....)))))......((((((.....))))))....))))).))))))))))).(((((.......(((....))).......))))).......((.(((.......))).)).((((.............))))...(.(((((....)))))).))))....))))).))
))))).((((.((....)).)))).)))))))..)))....))))).....))).))))))))))..)))(((((.((.((((...(((((((...(((.....))).))).((((....((((((((.........))))))))......)))).((.(((.((((((...((((
(..............(((.......))).......((((((.......)))))).....((((((....(((((...)))))........)))))).(((((.(((((((((((.((((..((.((((........)))).)).))))...))))))..((.((((((((((..((
((((......))))))....))..))..))))).)).))......)))))).))).)))))..))))))))).)).)..)))).((((((((((((((((((((..(((........((((.....))))......((.(((((((.........))))))))..))).))))))
)))))...........))))).)))............))))).)).))))..((((..((.(((.((((...(((((......))))))))).)))..)))))).))...))))).((((((...........)))))).((....))....)))))))))))......(((((..(((((.....
....))))))))))).((((((....))).)).........((((.........))))((((((............)))))......(((((......)))))...)))))))..........((((.(((((.((((((......)).)))))))))...))))....(((.........))
)..((((((....................))))))....((((..((...))..))))((((((........)))))..........(((((.(((....))).))))).((....))))).)).))))).((((.((((....))))))))..
```

Supplemental Figure S10

RNA 2

```
>ENERGY = -464.0  RNA2_no constrain
```

```
..((((.................(((((.........))))).((((.(((((.........))))).))))................((((((((..........(((((((((..((((..(((((..(((......))).)))))....))
))))))).)))))....))))).))))(((((.(((((...........)))))..((((((((.((((((((....)))).((((((..............((((((.(..((...(((((((((((((.((((...(((....)))......))
)).)))...)))))))))).))..)))))))..((((((((........((((.((((........((((((((.((((((((((((....))))))))))....))).))).))))))..((..(((((.(..........)))))..))...
((((((.(((.....)))))))))).)))).)))).)))))))))...))))))..((((((....((((((((....)))).))))((((((......))))))..))))))......)))))))))))))(((....((((.((.(((((((((..
...))))).)))))).))))...))).(((((((..(((..(((.........)))..)))..))))))).(((((.(.((.((((((.(((.........))).))))))....(((.((((((((((...)))))).)))).))).((((((...
)))))))((((.(((.((((((.(((((((((..((.((((..((((.....((.....))....)))).)))).))..)))))..........))))))).)))))).(((((.....(((((((((.....)).)))))))..))))).((((((((
((......)))).((((((((.(((......))).)))))))).(((............((((...((((((.((((((.(((..(((..((((.....))))..)))..))))....(((....))).(((.((((...(((((((((..((.((
((((((......(((....)))(((((.((((((......))))....))))))).))))))))))).))))......)))))..)))))))...)))).)).))))))..))))...))).))))))......(((..(((((.(((.((.....)).))
).))))))))....((.(((((.(((((....))))).))))).))........))))..(((((((...)))))))...)).)))))))...)))))......(((((....)))))......)))).((((....)))).....
```

```
>ENERGY = -424.1  RNA2_DMS-MaPseq corrected
```

```
((((((.....((((.((.....(((((.........))))).((((..((((.........))))..))))................((((((((..........(((((((((..((((..(((((..(((......))).)))))....))
))))))).)))))....))))).)))).((((((((((...........)))).))))))..........((((....)))).((((((..............((((((.(..((...(((((((((((((.((((...(((....)))......))
)).)))...)))))))))).))..)))))))..((((((((........((((.(((((..((((((((((((..((((.(((((((....)))))))....))))...))).))))).(((........)))......))))))..........
.(((((.(((.....)))))))).)))))).)))).))))))))...))))))..........((((((((....)))).)))).)).))))......))))))..........((((((((((((.....(((....((((.((.(((((((((..
...))))).)))))).))))...))).(((((((..(((..(((.........)))..)))..))))))).(((((.(((((((((((.(((.........))).))))))...(((((....((((((...)))).)).....((((((..(((((((
(.(((((.(((.(((.((((((.((((.((((..((.((((..((((.....((.....))....)))).)))).))..))...........))))))))).)))))).(((((.....((((((((.........)))))))..))))).......((
((......)))).((((((((.(((......))).))))))))..)))...)))))......)))))))...))))))).((.(((..(((..((((.....))))..)))..)))))((((((..((((.(((.((((...((((((..((..((.((
((((((......(((....)))(((((.((((((......))))....))))))).))))))))))).))........)))))..)))))))...))))....)))))))).)))))).))))))))).((((((((.......))))))))....(((((
.(((....((((..((((.(((.(((((....))))).))).(((.........))).((((((......)))))..........))))..)))).)))...)))))........))))))).)))))..((((....)))).....
```

Supplemental Figure S10. Dot-bracket maps of FHV RNA secondary structures.

DMS-MaPseq-imposed constrains allowed for substantial refolding and improvement over unconstrained thermodynamic predictions. The refolded nucleotides are highlighted in red.

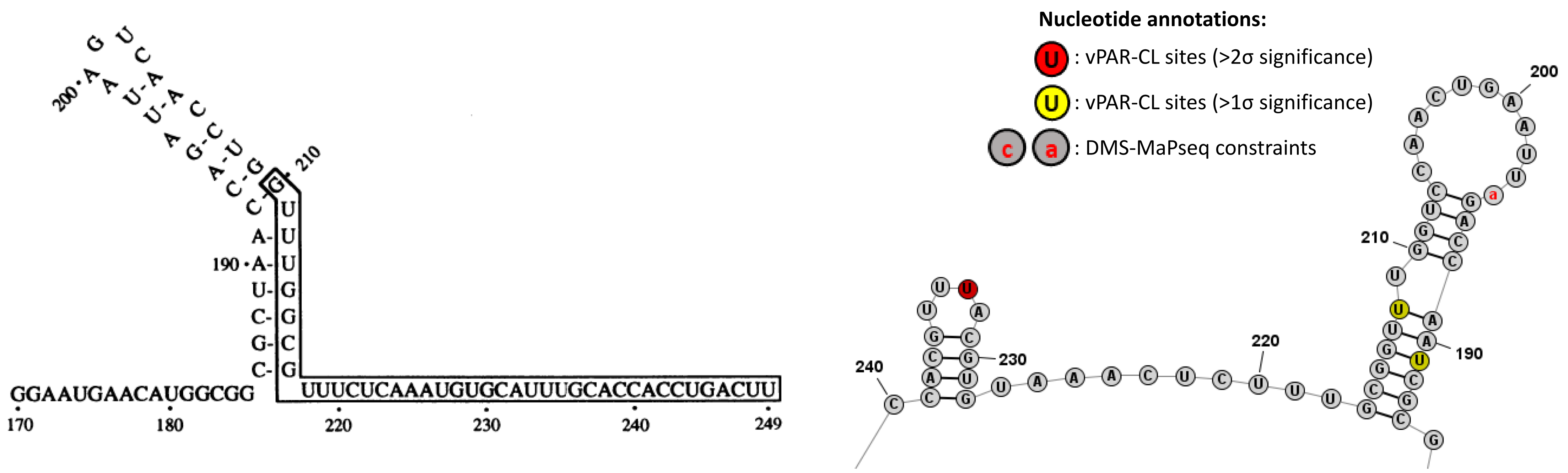

Supplemental Figure S11

Supplemental Figure S11. vPAR-CL and DMS-MaPseq predicted near identical structure to RNA 2 packaging signal.

A previously established RNA 2 packaging signal stem-loop structure was listed on the left. The stem loop and the boxed sequence (nt. 210-249) are determined to be essential for RNA 2 encapsidation (Zhong, et al., 1992. PNAS). Our DMS-MaPseq-imposed RNA structure is shown on the right, describing near identical structure. Three vPAR-CL sites were found in this region, explaining the importance of this site to RNA 2 packaing.

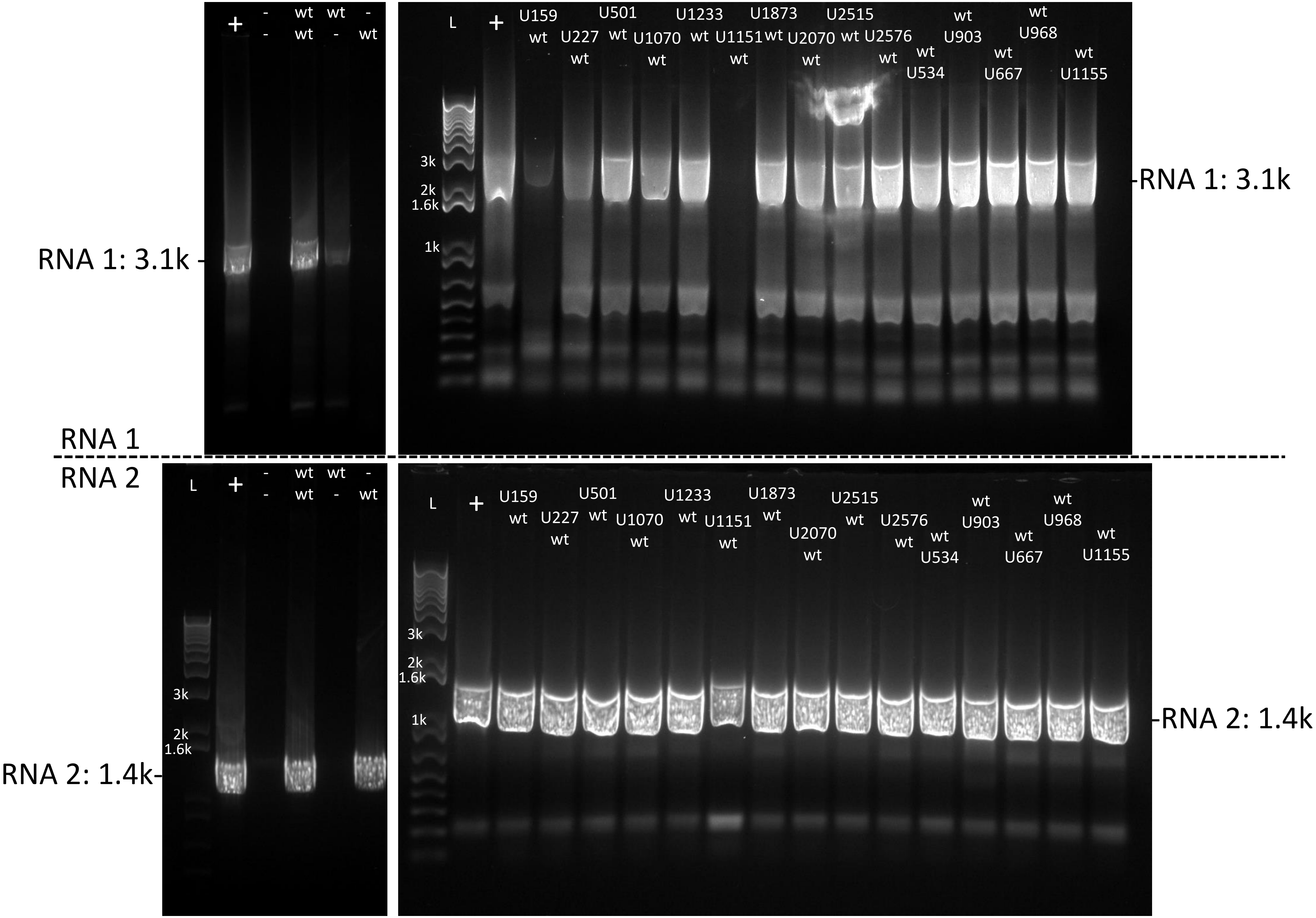

Supplemental Figure S12

Supplemental Figure S12. Original gel images for RT-PCR assays.

Original gel images for RT-PCR assays (Figure 7b) are provided. “+” denotes a control experiment where S2 cells were infected with wild type virions, while the rest represents S2 cells transinfected with FHV plasmids with/without mutations. L: 1kb plus DNA ladder (Invitrogen).

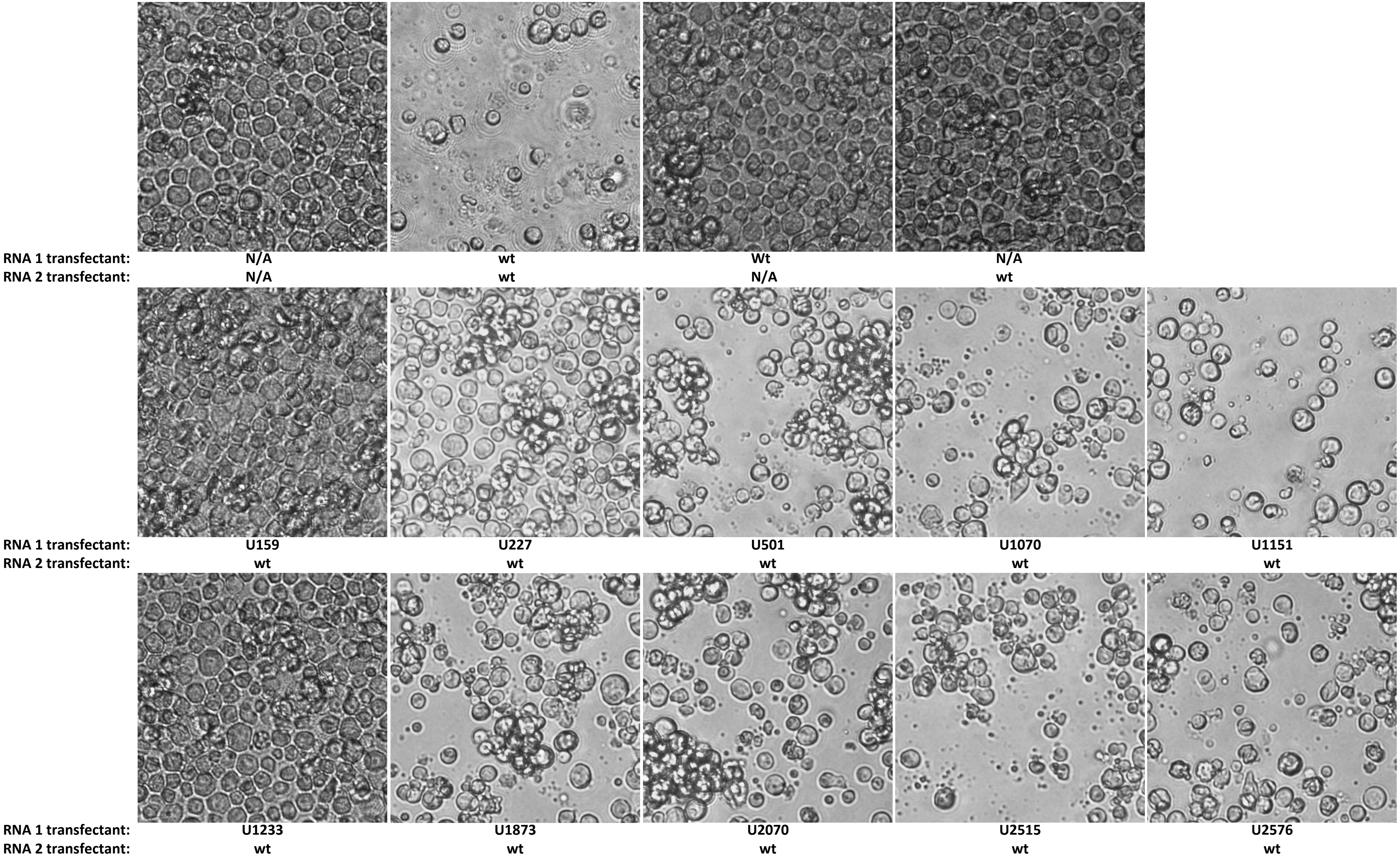

Supplemental Figure S13

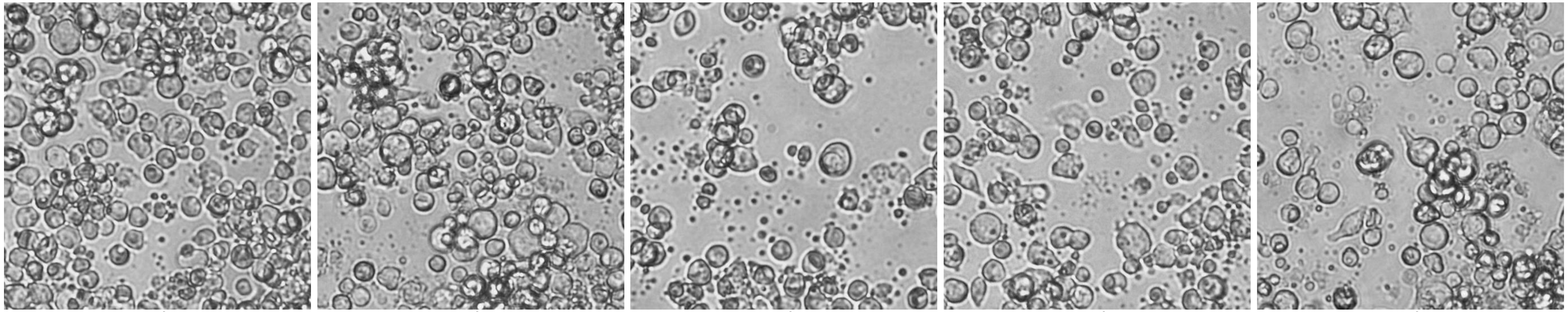

RNA 1 transfectant:	wt	wt	wt	wt	wt
RNA 2 transfectant:	U534	U667	U903	U968	U1155

Supplemental Figure S13 continued

Supplemental Figure S13. Bright field microscopy of FHV vPAR-CL mutants.

After generation of P0 mutant viruses, the P0 cell/virus mix was used to inoculate naïve S2 cells to yield P1 culture. Varied degrees of cytopathic effects (CPE) were observed with different mutants. U159, U1233 gave near wt appearance, indicating little to none virulence in these mutants. The rest mutants showed variable but inferior cytotoxic effect than wild type transfection.

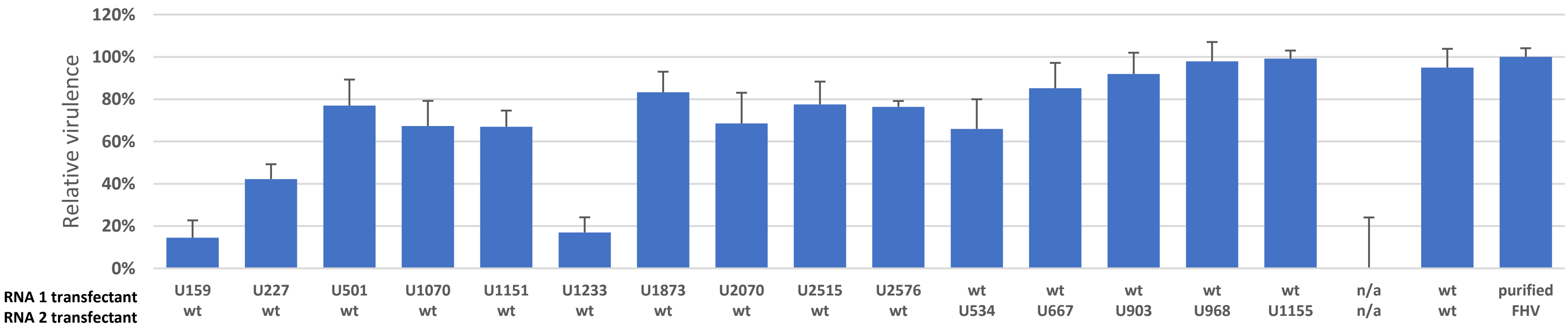

Supplemental Figure S14

Supplemental Figure S14. P1 vPAR-CL mutants and relative virulence.

Purified P1 vPAR-CL mutants were serial diluted and 0.12 ng of each mutant virus (approximately equivalent to MOI = 1) was used to infect naïve S2 cells (25k). AlamarBlue assay was conducted 24 h post infection. For negative control and mutants U157, U1233 with undetectable virus production, equal amount of cellular proteins from virus samples was used as guide.
