# Supplementary figures and images for: Mapping RNA–capsid interactions and RNA secondary structure within virus particles using next-generation sequencing
Source: Nucleic Acids Res. 2019 Dec 4;48(2):e12. doi: 10.1093/nar/gkz1124 (PMC6954446; doi:10.1093/nar/gkz1124)

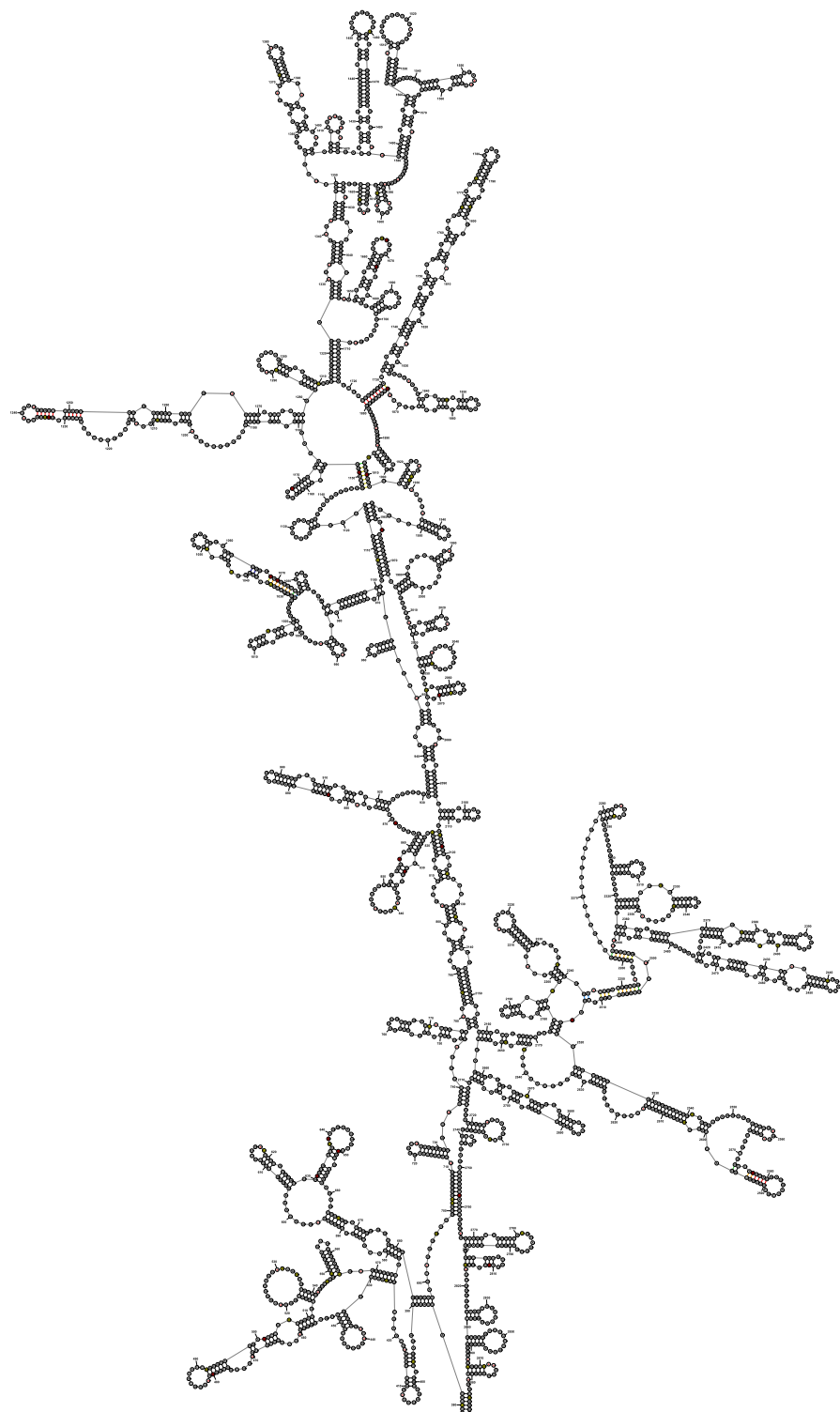

Supplement: gkz1124_Supplemental_Files [file gkz1124_supplemental_files.zip › Supplemental data 1.pdf]

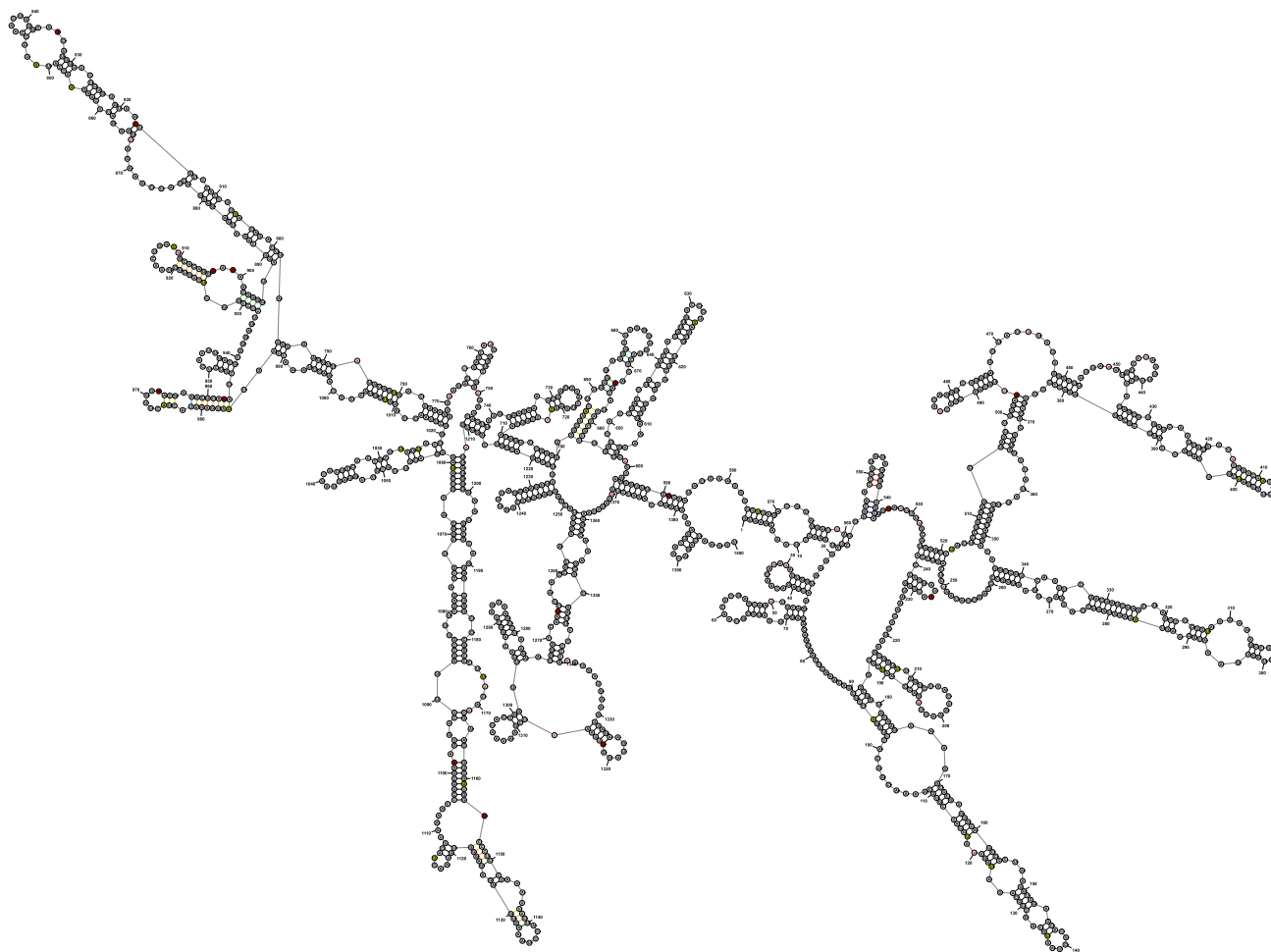

Supplement: gkz1124_Supplemental_Files [file gkz1124_supplemental_files.zip › Supplemental data 2.pdf]
